# Ring Formation and Hydration Effects in Electron Attachment to Misonidazole

**DOI:** 10.3390/ijms20184383

**Published:** 2019-09-06

**Authors:** Milan Ončák, Rebecca Meißner, Eugene Arthur-Baidoo, Stephan Denifl, Thomas F. M. Luxford, Andriy Pysanenko, Michal Fárník, Jiří Pinkas, Jaroslav Kočišek

**Affiliations:** 1 Institut für Ionenphysik und Angewandte Physik, Leopold-Franzens Universität Innsbruck, Technikerstrasse 25, Innsbruck A-6020, Austria (R.M.) (E.A.-B.) (S.D.); 2 Atomic and Molecular Collisions Laboratory, CEFITEC, Department of Physics, Universidade Nova de Lisboa, 2829-516 Caparica, Portugal; 3 Center for Biomolecular Sciences Innsbruck, Leopold-Franzens Universität Innsbruck, Technikerstrasse 25, Innsbruck A-6020, Austria; 4 J. Heyrovský Institute of Physical Chemistry v.v.i., The Czech Academy of Sciences, Dolejškova 3, 18223 Prague, Czech Republic (T.F.M.L.) (A.P.) (M.F.) (J.P.)

**Keywords:** misonidazole, clusters, low-energy electron, bond formation, electron attachment

## Abstract

We study the reactivity of misonidazole with low-energy electrons in a water environment combining experiment and theoretical modelling. The environment is modelled by sequential hydration of misonidazole clusters in vacuum. The well-defined experimental conditions enable computational modeling of the observed reactions. While the NO${}_{2}^{-}$ dissociative electron attachment channel is suppressed, as also observed previously for other molecules, the OH${}^{-}$ channel remains open. Such behavior is enabled by the high hydration energy of OH${}^{-}$ and ring formation in the neutral radical co-fragment. These observations help to understand the mechanism of bio-reductive drug action. Electron-induced formation of covalent bonds is then important not only for biological processes but may find applications also in technology.

## 1. Introduction

Low-energy electrons, which can be formed as secondary species after the interaction of radiation with living matter, are well known reactive species. Reactions of low-energy electrons with DNA can result in severe damage [1], especially when taking into account their large quantity [2,3] and different processes of their formation [4]. The most studied process of DNA damage by low-energy electrons is dissociative electron attachment (DEA):

$$\mathrm{AB}+e^{-}\to A+B^{-}$$

Radiation damage by DEA is unique, in that it can break covalent bonds at sub-excitation energies. Even hydrated electrons, with solvation free energies of ∼1.5 eV [5], can break the bonds if the energy gained due to electron affinity of one of the fragments is enough to overcome the dissociation barriers. This unique feature of DEA was proposed to be a key for the development of novel radiosensitizers—molecules enhancing the combined action of concurrent chemo-radiation treatment of tumors [6]. However, other processes induced by low-energy electrons may also be important in this manner, including inelastic electron scattering [3] and associative electron attachment [7,8]. Identification of the processes importance in radiosensitization requires systematic studies of DEA to molecules with known radiosensitizing effects. An example of such a molecule is misonidazole ((RS)-1-Methoxy-3-(2-nitroimidazol-1-yl)propan-2-ol, MISO), which is studied in the present work.

Misonidazole is a prototypical system of a bio-reductive agent. The bio-reductivity can be used for targeted action of the molecule in a hypoxic environment [9] in imaging [10,11] or radiosensitizing [12,13] applications. The bio-reduction may work on several different time scales. On the shortest scale, a single electron reduction can occur as known for other nitro substituted compounds [14] and as was recently also proposed for nimorazole [7]. The single electron reduction may be important in radiosensitization occurring immediately after irradiation [15,16] or for molecular transport within the cell structures [7]. On longer timescales, reduction by several electrons can occur, which results in formation of free radicals, radical anions or complex metabolites with DNA segments [17]. Most of these reduction products were, however, shown to be further biologically inactive [18].

In this work, we focus on the single electron reduction and processes that immediately follow this step in a water environment. The study was performed under vacuum conditions, colliding free electrons with model clusters consisting of MISO and a controlled number of water molecules. These well-defined experimental conditions enable us to experimentally study the DEA energetics and perform computational modelling of the processes immediately following the dissociation. The study is a continuation of our systematic exploration of low-energy electron induced chemistry of nitro-imidazolic radiosensitizers [19,20]. In the present issue, we also report on the electron induced chemistry of isolated MISO [21].

We show that while the nitro group dissociation is suppressed upon the hydration of MISO, the hydroxyl group dissociation channel remains open in a water environment. A reasonable explanation for the hydroxyl group dissociation for low-energy incident electrons is based on the formation of a covalent C-N bond following the DEA. The structures enabling covalent bond formation and synthesis under the action of low-energy electrons attracted significant interest in recent years due to possible technological applications and understanding of fundamental astrochemical reaction mechanisms [22,23,24]. Apart from a very low energy input for inducing the reaction, also the site selectivity and possible catalytic action makes the low-energy electron an attractive trigger of reactivity [25]. The formation of C-N bonds under the action of low-energy electrons was formerly predicted also for azabenzene.(CO${}_{2}$)${}_{n}$ clusters [26]. Here, we show that the C-N bond formation occurs on a single molecule and creates a neutral cyclic π-bonded system.

## 2. Materials and Methods

### 2.1. Experiment

We used a source of microhydrated clusters [27] and a reflectron time-of-flight mass spectrometer (RTOF) [28], which are parts of the complex CLUster Beam (CLUB) setup [29]. The beam of microhydrated MISO was prepared by co-expanding a mixture of a buffer He or Ne gas, humidified by the Pergo gas humidifier system, together with sublimed MISO (98% purity, Toronto Research Chemicals) through a 90 μm nozzle into vacuum (10${}^{-4}$ mbar range during the experiment). The beam was skimmed and, 1.5 m downstream, it entered the interaction region of the RTOF (10${}^{-8}$ mbar during the experiment) where it was crossed by a beam of low-energy electrons. The electrons were produced by thermal emission from a tungsten cathode with an energy distribution width of ∼0.7 eV. Electrons were then accelerated to the required kinetic energy in the range of 0.6–5.6 eV in the interaction region. Product anions were extracted directly from the interaction zone into the RTOF where they were separated according to their mass to charge ratio. The ion signal was acquired using a time to digital conversion method.

The MISO sublimation temperature was 363 K, which is high for a biomolecule. Therefore, we checked thermal decomposition of the molecule by prolonged heating (8 h) at an elevated temperature (390 K) and NMR analysis of the original and heated sample. The NMR analysis of both samples (as solutions in DMSO-d${}_{6}$) showed nearly identical spectra, indicating a reasonable thermal stability of MISO.

### 2.2. Theory

The MISO.(H${}_{2}$O)${}_{n}$ clusters and their fragments were first optimized at the B3LYP/6-31+g* level of theory along with D2 dispersion correction as proposed by Grimme [30]. The resulting structures were then re-optimized at both B3LYP+D2/aug-cc-pVDZ and M06/aug-cc-pVDZ levels of theory to assess the error of calculations. For scanning possible fragment isomers, we used molecular dynamics at the semi-empirical PM6 level and at various temperatures, with a time step of 40 a.u. (∼0.96 fs). The Gaussian program [31] was used for all quantum chemical calculations reported, molecular dynamics was performed in the Abin code [32]. Supplementary material: Cartesian coordinates of structures optimized in the present study.

## 3. Results and Discussion

### 3.1. Molecular Fragmentation

**Figure 1:**
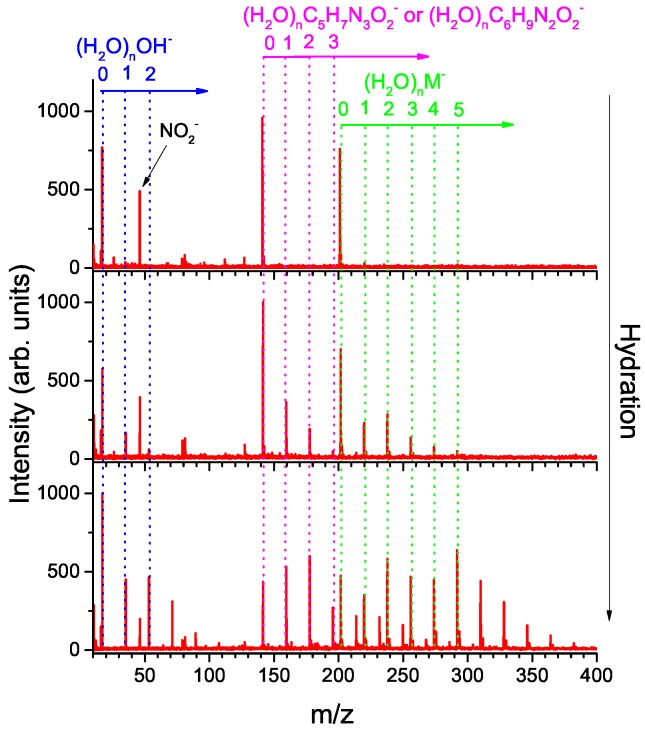
Cumulative mass spectra for negative ion formation after interaction of electrons in the 0.6–5.6 eV range with MISO in a molecular beam without hydration and at two different levels of hydration. The number of water molecules attached to misonidazole in the neutral precursor cluster increases from top to bottom.

**Figure 2:**
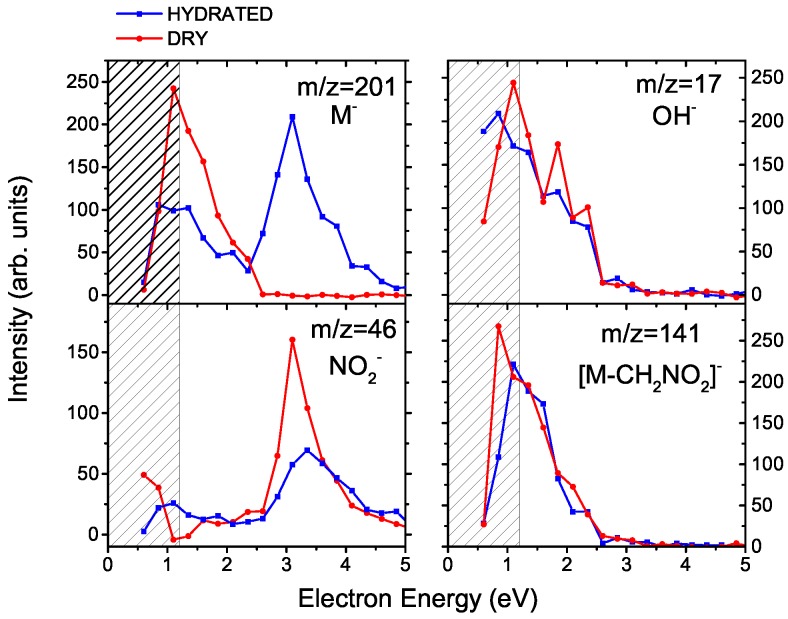
Ion yields for the formation of selected anions from misonidazole as a function of the energy of the incident electron. Red and blue curves show a molecular beam of MISO without and with hydration, respectively (“dry” and “hydrated”). The region of a strong decrease in the electron current is hatched.

Anion mass spectra for MISO at different hydration conditions are depicted in Figure 1. The anion formation has a resonant character as can be seen from Figure 2, which shows electron energy dependent ion yields. Therefore, we are showing cumulative spectra, which are obtained by summing individual spectra taken at energies ranging from 0.6 to 5.6 eV with a step of 0.25 eV.

The top panel of Figure 1 represents “dry” conditions when pure He, without humidification, was used as a buffer gas. These data may be compared to the results for isolated MISO [21] (see Table 1). Isolated MISO fragments primarily to m/z=46 (NO${}_{2}^{-}$; 100), m/z=201 (M${}^{-}$; 50) and m/z=141 (25) anions, with numbers in parentheses representing the integrated yield of the anion. In the present experiment, the four most intense anions are m/z=141 (100), M${}^{-}$(75), NO${}_{2}^{-}$(30), OH${}^{-}$(22). The m/z=141 anion can be assigned to either [MISO-CH${}_{2}$NO${}_{2}$]${}^{-}$ or [MISO-C${}_{2}$H${}_{4}$O${}_{2}$]${}^{-}$ as discussed below.

In the present molecular beam experiment at dry conditions, we observe (i) lower relative intensity of the m/z=46 anions; (ii) higher relative intensity of m/z=141 anions; (iii) OH${}^{-}$ anions that have not been investigated for the isolated molecule. There are two possible reasons for the observed differences:

(i) *Differences in the experimental approaches.*

The first difference is the used mass spectrometer. Transmission efficiency of the quadrupole mass spectrometer, used in the isolated molecule study, may be lower for high mass fragments.

Second, the electron source in Ref. [21] is dedicated to electron attachment spectroscopy, with excellent performance at low electron energies. The simple electron gun at the CLUB setup cannot produce reliable results at electron energies below ∼1.2 eV [33], this region is therefore hatched in Figure 2. We can see that this discrepancy may result in lowering the anion signal of resonances at incident electron energies near 0 eV.

The third important difference is in the used molecular beams, which was effusive in the gas phase study and adiabatic expansion in the present experiment. In the beam experiment, the sublimed molecules are cooled down by buffer gas collisions, which may result in much lower neutral precursor temperature during the electron attachment. This may be a reason for the higher parent anion M${}^{-}$ signal in the present experiments, despite the mentioned low efficiency of our electron gun at low electron energies.

(ii) *Different neutral precursor.*

We analyzed the sample by ${}^{1}$H NMR spectroscopy in DMSO-d${}_{6}$. Besides the MISO signals, we have found low-abundant signals of byproducts (in total ca 1 mol % with respect to MISO, which is consistent with stated purity of the sample, 98%). Among them, 1-ethyl-2-nitroimidazole (around 0.5 mol % with respect to MISO) was determined as the main species. The ${}^{1}$H NMR spectrum showed a triplet at 1.39 and quartet at 4.40 ppm (with a mutual coupling constant ${}^{3}$J${}_{HH}$ = 7.2 Hz), corresponding to the methyl and methylene groups of the ethyl substituent. In addition, heterocycle protons were found at 7.22 and 7.87 ppm, respectively. These signals correspond to previously published data for 1-ethyl-2-nitroimidazole and its derivatives [34]. The mass of the 1-ethyl-2-nitroimidazole is 141, therefore the m/z=141 anion may be formed by direct electron attachment to a neutral 1-ethyl-2-nitroimidazole molecule. Despite its low mol % in the solid sample, the higher vapour pressure of 1-ethyl-2-nitroimidazole could lead to a higher partial pressure in the gas phase, explaining the intense peak observed in the cumulative mass spectra.

**Figure 3:**
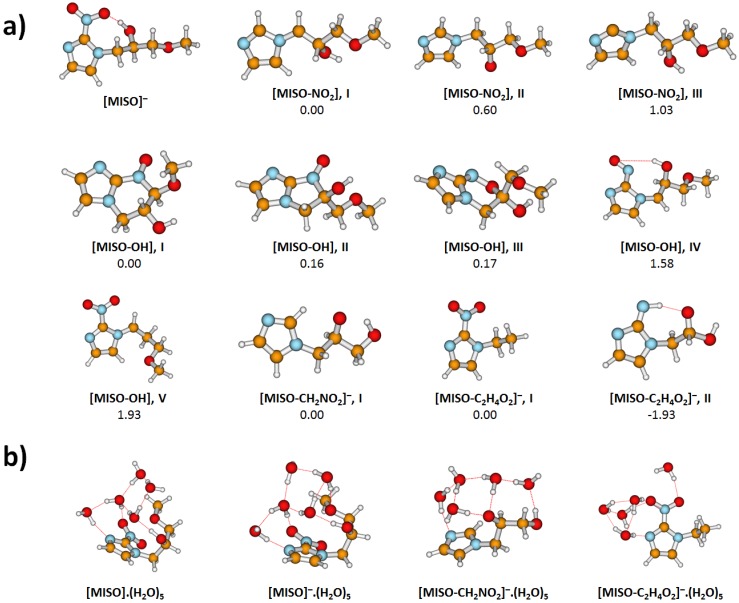
Optimized structures of MISO clusters and dissociation fragments, calculated at the B3LYP+D2/aug-cc-pVDZ level. (**a**) Various isomers of non-hydrated dissociation products, along with relative energy (in eV). (**b**) Selected hydration structures for five water molecules.

The 1-ethyl-2-nitroimidazole could be a sample impurity or it could be formed by thermal decomposition of MISO. This is suggested by our theoretical calculations, showing that a strongly exothermic channel exists, where MISO decomposes into a neutral fragment with the mass of 141, which is the neutral analogue of isomer **I** in Figure 3. The corresponding reaction is (as calculated at the B3LYP+D2/aug-cc-pVDZ level of theory):

$$\left(N1\right)\;\mathrm{MISO}\to \left[\mathrm{MISO}-C_{2}H_{4}O_{2}\right]+{\mathrm{CH}}_{4}+{\mathrm{CO}}_{2}+1.32\;\mathrm{eV}$$

Note that the respective energy might reach up to 2.2 eV if a ring is formed. The fact that this channel may be a thermal decomposition product of the sample does not decrease its importance for the combined chemo-radiation therapy. Activation barriers for the decomposition may be easily overcome by the action of ionizing radiation [35]. Also, thermal and photothermal therapies are of increasing interest [36]. However, as already stated, the comparison of ${}^{1}$H NMR spectra of the sample before and after heating is not significantly different, which may indicate that the decomposition products, including the 1-ethyl-2-nitroimidazole, sublime at the decomposition temperature.

Several more fragment anions with low intensities were observed for the isolated molecule in [21], below the detection sensitivity of our instrument. Only m/z=26,97 and 112 anions are revealed in the present spectra.

### 3.2. Water Solvent Effects

We will focus here on the effect of the water solvent on the main dissociation channels. Examples of mass spectra obtained for two hydration conditions are shown in the middle and bottom panel of Figure 1.

First, we can see that M${}^{-}$, [MISO-CH${}_{2}$NO${}_{2}$]${}^{-}$/[MISO-C${}_{2}$H${}_{4}$O${}_{2}$]${}^{-}$ and OH${}^{-}$ bind strongly to water as revealed by the presence of hydrated clusters in the spectrum. Particularly interesting are the intensity enhancements for the (H${}_{2}$O)${}_{n}$M${}^{-}$ anions with n=2 and n=5 or a significant intensity drop for (H${}_{2}$O)${}_{n}$[M-CH${}_{2}$NO${}_{2}$]${}^{-}$/[MISO-C${}_{2}$H${}_{4}$O${}_{2}$]${}^{-}$ anions above n=2.

**Figure 4:**
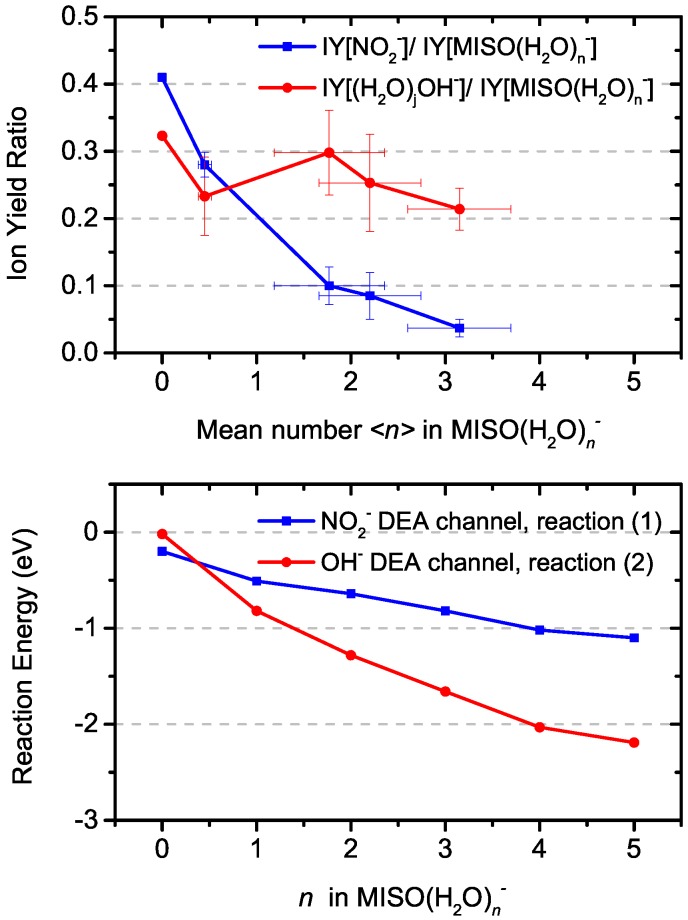
Evolution of relative ion yields for OH${}^{-}$ and NO${}_{2}^{-}$ fragments as a function of hydration (**top**) and computed reaction energies for respective DEA reaction channels (**bottom**).

Generally, a water solvent reduces DEA fragmentation of biomolecules [7,8,27]. This effect can be seen also for the NO${}_{2}^{-}$ channel from MISO in Figure 4, which shows that the branching ratio of the NO${}_{2}^{-}$ fragment ions with respect to the total intensity of (H${}_{2}$O)${}_{n}$M${}^{-}$ decreases at higher levels of hydration. The decrease of the fragmentation is not as steep as for the previously studied radiosensitizer nimorazole [7]. If we compare the decrease for hydration conditions characterized by in average n=3 water molecules in (H${}_{2}$O)${}_{n}$M${}^{-}$ hydrated parent anion clusters, the NO${}_{2}^{-}$ signal decreases 10 times in comparison to the parent ion signal while the decrease in the case of nimorazole is 100 times. The observation may be influenced by a different number of water molecules evaporating from the cluster after electron attachment and, consequently, different neutral precursor cluster sizes. We have shown that the number of evaporated water molecules depends on the adiabatic electron affinity of the molecule [8]. The adiabatic electron affinity of nimorazole is ∼ 1.3 eV [7] and that of MISO is 1.33 eV [37]. Therefore, we do not expect significant differences in the hydration levels of the two molecules.

The slowly disappearing NO${}_{2}^{-}$ fragmentation channel will probably not be closed completely in solution and may be the cause of the higher toxicity of MISO in comparison to nimorazole [38]. The interconnection of DEA to biological activity and toxicity has been reviewed recently [39].

The closing of the NO${}_{2}^{-}$ DEA channel can be observed also in the energy dependent ion yields, shown at the left panels of Figure 2. After hydration, the NO${}_{2}^{-}$ total intensity decreases and a resonance at ∼3 eV starts to appear in the spectrum of the parent anion clusters.

In contrast to the NO${}_{2}^{-}$ signal, intensity of the OH${}^{-}$ fragment seems to be independent of hydration. While there may be some decrease, this decline lies within the error bars of the present experiment. The (H${}_{2}$O)${}_{n}$OH${}^{-}$ signal is not caused by direct electron attachment to water, as water does not have any low-lying DEA resonances [40]. Also, the OH${}^{-}$ signal has not been observed after electron attachment to other types of clusters of water with biological molecules [27] or nitro compounds [7]. On the other hand, a similar behavior was observed for deoxycytidine monophosphate [41]. In the following section, we will show that the OH${}^{-}$ release in water environment is caused by a large hydration energy of OH${}^{-}$ and illuminate the complex process that drives its dissociation from MISO at low energies.

### 3.3. Theoretical Model for DEA from MISO

We performed quantum chemical calculations of gas phase and hydrated molecules and ions, with results shown in Figure 3 and Figure 4 and Table 2.

**Figure 5:**
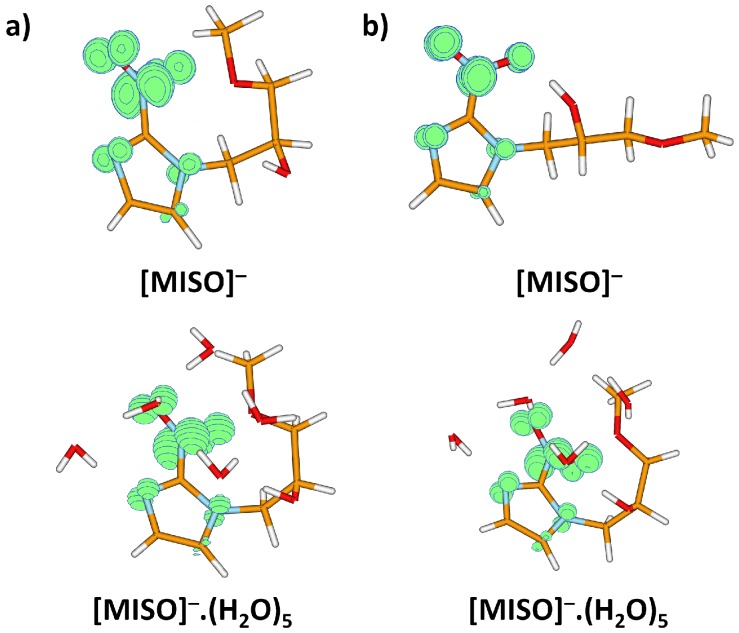
Spin density for the misonidazole anion (**a**) in the structure of the neutral molecule; (**b**) for the minimum located after optimization in the anionic state. Both MISO${}^{-}$ and MISO${}^{-}$.(H${}_{2}$O)${}_{5}$ were considered. Calculated at the B3LYP+D2/aug-cc-pVDZ level.

After electron attachment, the electron is located at the NO${}_{2}$ group, see Figure 5. Starting with the vertical electron affinity (VEA), it can be seen that water molecules stabilize the anionic state more than the neutral one, from 0.84 eV for MISO to about 1.4 eV for MISO.(H${}_{2}$O)${}_{5}$. The adiabatic electron affinity is by about 0.6 eV higher, with the reorganization energy mainly accounting for more efficient hydration of the negatively charged NO${}_{2}$ group. For example, an OH...NO${}_{2}$ hydrogen bond is formed in [MISO]${}^{-}$, see Figure 3. However, the spin density distribution stays very similar (Figure 5). Among possible dissociative channels, we considered the following reactions:

(1) MISO${}^{-}$.(H${}_{2}$O)${}_{n}\to$ NO${}_{2}^{-}$.(H${}_{2}$O)${}_{n}$ + [MISO-NO${}_{2}$]
(2) MISO${}^{-}$.(H${}_{2}$O)${}_{n}\to$ OH${}^{-}$.(H${}_{2}$O)${}_{n}$ + [MISO-OH]
(3a) MISO${}^{-}$.(H${}_{2}$O)${}_{n}\to$ [MISO-CH${}_{2}$NO${}_{2}$]${}^{-}$.(H${}_{2}$O)${}_{n}$ + CH${}_{2}$NO${}_{2}$
(3b) MISO${}^{-}$.(H${}_{2}$O)${}_{n}\to$ [MISO-C${}_{2}$H${}_{4}$O${}_{2}$]${}^{-}$.(H${}_{2}$O)${}_{n}$ + CH${}_{3}$OH + CO

The NO${}_{2}^{-}$ channel (1) produces a [MISO-NO${}_{2}$] radical. If a simple NO${}_{2}$ dissociation is considered, isomer **III** in Figure 3 is formed. However, the carbon atom to which the NO${}_{2}$ group was connected now carries the odd electron. If a proton is transferred from the CH${}_{2}$ group attached to the imidazole ring (isomer **I**), stabilization by about 1 eV is observed, as already noted elsewhere [21].

In the case of the OH${}^{-}$ channel (2), the situation is more complicated. Here, a simple dissociation reaction leads to a high-lying [MISO-OH] isomer, making reaction (2) endothermic by about 2 eV, isomer **V**. The dissociation energy can be reduced by about 0.4 eV when the dissociating oxygen comes from the NO${}_{2}$ group, isomer **IV**. However, the most stable structures found for [MISO-OH] are the ones including ring formation, e.g., isomers **I-III**. The most stable configuration found has a six-membered ring (**I**), with a structure with a five-membered ring being close in energy (**II**). The respective rings can be formed with only small structural rearrangements from isomer **IV**. Only when the ring formation is accounted for, the low energy of the respective resonances in Figure 2 can be explained.

The stochiometry of m/z=141 can correspond either to [MISO-CH${}_{2}$NO${}_{2}$]${}^{-}$ or [MISO-C${}_{2}$H${}_{4}$O${}_{2}$]${}^{-}$. In both cases, a considerable structural rearrangement is needed to form the respective anion. In the first case, the NO${}_{2}$ group along with a CH${}_{2}$ from the alkyl chain has to dissociate, leading to an overall endothermic reaction (3a) with the reaction energy of about 0.4 eV. In the second case, CH${}_{3}$OH and CO might dissociate from the imidazole substituent after substantial rearrangement. In this case, however, an exothermic reaction with the energy of about −0.8 eV is predicted. Note that even a more stable isomer **II** can be formed, its formation would however require more substantial changes in bonding. At the same time, about 1.7 eV more energy can be obtained if CH${}_{4}$ and CO${}_{2}$ dissociate instead of CH${}_{3}$OH and CO, making the reaction exothermic even in the neutral state (see Equation (N1)).

The m/z=141 anions can be therefore formed by direct electron attachment to the m/z=141 impurity of the sample (0.5% mol) or as a thermal decomposition product of MISO or by DEA to MISO. In all cases, the most probable structure is the 1-ethyl-2-nitroimidazole anion ([MISO-C${}_{2}$H${}_{4}$O${}_{2}$]${}^{-}$, **I** depicted in Figure 3).

In the gas phase, reaction (3b) is the only one that is markedly exothermic, reactions (1) and (2) are almost thermoneutral and reaction (3a) is endothermic. Upon hydration, reaction (2) is most markedly influenced due to the efficient hydration of OH${}^{-}$ compared to other ions and becomes more exothermic than reaction (1) already for hydration with one water molecule. This can explain the experimentally observed increase in the OH${}^{-}$/NO${}_{2}^{-}$ ratio in Figure 4. Reactions (3a,b) are less influenced, with each water molecule shifting the DEA energy by about 0.2 eV.

Let us stress here that energy gained by hydration of OH${}^{-}$ is characteristics of hydroxyl anion, independent of the precursor molecule. This energy gain may therefore enable dissociation after electron attachment in water environment to many other molecules containing hydroxyl functional groups.

## 4. Conclusions

We have demonstrated how the molecular environment influences interactions of low-energy electrons (0–5 eV) with a model bio-reductive therapeutics molecule, MISO. Negative ion mass spectra show the suppression of fragmentation, except for the OH${}^{-}$ dissociation channel. Quantum chemical calculations show that hydration of OH${}^{-}$ is much more energetically favourable than hydration of the proposed NO${}_{2}^{-}$ reaction byproducts. The mechanism may be important also in other OH-containing biomolecules, such as the previously studied dCMP [41]. At the same time, the reaction energy of the simple OH${}^{-}$ dissociation is high and only the formation of a new covalent bond in the neutral by-products may explain its observation at very low electron energies. Here, the most probable ring formation occurs after a loss of an oxygen atom from the nitro group and subsequent formation of a new C-N bond. The proposed behavior may be tested on further nitro- compounds substituted by long hydrocarbon chains.

## Figures and Tables

**Table 1:** Relative ion yields for the main anions observed after electron attachment to MISO integrated over the studied electron energy range. (i) isolated molecule from Ref. [21], (ii) expansion without hydration and (iii) highest hydration conditions. The values are scaled to 100 for the most intense ion yield, m/z=141 fragment may result from sample impurity. The ion yields at hydrated conditions are sums of yields of anion and its hydrated clusters.

m/z	Ion	Relative Ion Yield		
		(i) isolated [21]	(ii) dry	(iii) hydrated
201	MISO${}^{-}$	50	75	100
141	[MISO-CH${}_{2}$NO${}_{2}$]${}^{-}$ or [MISO-C${}_{2}$H${}_{4}$O${}_{2}$] ${}^{-}$	25	100	50
46	NO${}_{2}^{-}$	100	30	3
17	OH${}^{-}$	-	22	20

**Table 2:** Reaction energies (in eV) for electron attachment and DEA reactions for MISO(H${}_{2}$O)${}_{n}$ clusters in dependence on the number of hydrated water molecules. Reaction energies are given with respect to isomers **I** shown in Figure 3. Calculated at the B3LYP+D2/aug-cc-pVDZ (M06/aug-cc-pVDZ) level.

*n*	VEA	AEA	R. (1)	R. (2)	R. (3a)	R. (3b)
0	0.84 (0.81)	1.45 (1.42)	−0.20 (−0.06)	−0.02 (0.13)	0.41 (0.49)	−0.83 (−0.76)
1	1.01 (0.96)	1.63 (1.66)	−0.51 (−0.43)	−0.82 (−0.73)	0.05 (0.23)	−1.09 (−1.05)
2	1.09 (0.97)	1.84 (1.77)	−0.64 (−0.49)	−1.28 (−1.13)	−0.12 (0.07)	−1.33 (−1.23)
3	1.22 (1.19)	2.07 (1.99)	−0.82 (−0.70)	−1.66 (−1.55)	−0.27 (−0.09)	−1.45 (−1.33)
4	1.48 (1.45)	2.17 (2.08)	−1.02 (−0.92)	−2.03 (−1.94)	−0.62 (−0.41)	−1.71 (−1.61)
5	1.35 (1.33)	2.14 (2.06)	−1.10 (−1.04)	−2.19 (−2.14)	−0.58 (−0.32)	−1.81 (−1.71)

## Supplementary Materials

Supplementary materials can be found at https://www.mdpi.com/1422-0067/20/18/4383/s1.

## Abbreviations

The following abbreviations are used in this manuscript:

DEA	Dissociative Electron Attachment
RTOF	Reflectron Time of Flight Mass Spectrometer
MISO	Misonidazole
DMSO	dimethyl sulfoxide
