# Supplementary material for: Ring Formation and Hydration Effects in Electron Attachment to Misonidazole
Source: Int J Mol Sci. 2019 Sep 6;20(18):4383. doi: 10.3390/ijms20184383 (PMC6770096; doi:10.3390/ijms20184383)
Supplement: Supplementary file 1 [file ijms-20-04383-s001.pdf]

Supporting Information

for

Ring Formation and Hydration Effects in Electron Attachment to Misonidazole

Milan Ončák,[1,*] Rebecca Meißner,[1,2,3] Eugene Arthur–Baidoo,[1,2] Stephan Denifl,[1,2] Thomas F. M. Luxford,[3] Andriy Pysanenko,[3] Michal Fárník,[3] Jiří Pinkas,[3] and Jaroslav Kočišek[3,*]

[1] Institut für Ionenphysik und Angewandte Physik, Leopold-Franzens Universität Innsbruck, Technikerstrasse 25, A-6020, Innsbruck, Austria

[2] Center for Biomolecular Sciences Innsbruck, Leopold-Franzens Universität Innsbruck, Technikerstrasse 25, A-6020, Innsbruck, Austria

[3] Atomic and Molecular collisions Laboratory, CEFITEC, Department of Physics, Universidade Nova de Lisboa, 2829-516 Caparica, Portugal

[4] J. Heyrovský Institute of Physical Chemistry v.v.i., The Czech Academy of Sciences, Dolejškova 3, 18223 Prague, Czech Republic

* Correspondence: Milan.Oncak@uibk.ac.at (M.O.); kocisek@jh-inst.as.cz (J.K.)

Cartesian coordinates of structures optimized at the B3LYP+D2/aug-cc-pVDZ level (in Å) along with zero-point corrected energies (in Hartree)

MISO
E= -738.331392
C 1.885467 -1.897099 -0.999211
N 2.215278 -0.598525 -0.752414
C 1.333387 -0.188869 0.135797
N 0.434840 -1.161981 0.497831
C 0.781287 -2.259865 -0.244686
N 1.314737 1.157337 0.648423
O 1.989097 1.997820 0.070207
C -0.777656 -1.114171 1.322242
C -2.031272 -0.675984 0.539924
C -2.034281 0.789723 0.114158
O -1.080206 0.950938 -0.924795
C -0.950443 2.305894 -1.340791
O 0.611259 1.386920 1.643542
O -2.266560 -1.542421 -0.563307
H 0.211838 -3.179084 -0.174125
H 2.452693 -2.508671 -1.693510
H -0.942104 -2.138106 1.676780
H -0.603725 -0.453388 2.173018
H -2.875815 -0.817485 1.230751
H -1.724887 -1.224234 -1.299604
H -1.778475 1.430176 0.974757
H -3.039520 1.059942 -0.255902
H -0.200397 2.325028 -2.139557
H -1.914317 2.690904 -1.718139
H -0.606932 2.933269 -0.501122

MISO.H2O
E= -814.769506
C -2.058263 -1.244197 -0.301557
C -2.770813 -0.434730 0.569278
N -2.213365 0.810389 0.612485
C -1.196082 0.753128 -0.223082
N -1.053685 -0.473965 -0.820468
N -0.341927 1.885609 -0.489786
O 0.364044 1.848883 -1.506239
C -0.007918 -1.013260 -1.697638
C 1.208931 -1.580282 -0.936428
O 0.830864 -2.593160 -0.031336
O -0.365703 2.813702 0.305920
C 2.146556 -0.522413 -0.353536
O 1.530494 0.218829 0.697857
C 2.333446 1.319572 1.124292
H -2.156044 -2.287427 -0.576945
H -3.653019 -0.685856 1.149301
H -0.474061 -1.841709 -2.242177
H 0.297919 -0.236271 -2.400017
H 1.809677 -2.078031 -1.715329
H 0.447852 -2.205558 0.785183
H 2.434670 0.172938 -1.160466
H 3.049989 -1.023010 0.034206
H 1.796586 1.808910 1.944419
H 3.318485 0.965636 1.472599
H 2.462731 2.031464 0.293329
O -0.054471 -1.320990 2.288488
H 0.507508 -0.600530 1.941560
H -0.956423 -0.979812 2.233232

MISO.2H2O
E= -891.206923
N 0.226484 -1.244463 -0.989730
C -0.106516 -2.446207 -0.427302
C -1.444917 -2.369212 -0.083482
N -1.934134 -1.138085 -0.402458
C -0.912850 -0.492667 -0.937934
N -1.018379 0.861550 -1.410303
O -1.963071 1.530219 -0.999899
C 1.607015 -0.884264 -1.329925
C 2.375387 -0.252772 -0.144605
C 2.040037 1.215060 0.105985
O 0.730646 1.330244 0.634482
C 0.371082 2.681937 0.868456
O -0.163699 1.268775 -2.202807
O 2.286091 -1.054230 1.008420
O 0.055084 -0.694221 2.581729
H 0.637168 -3.222494 -0.298231
H -2.067462 -3.130651 0.373934
H 2.101272 -1.826111 -1.592288
H 1.598960 -0.219151 -2.195453
H 3.431103 -0.264663 -0.462568
H 1.460258 -0.856323 1.507578
H 2.119698 1.775596 -0.843845
H 2.767826 1.627034 0.827604
H -0.602149 2.676077 1.372101
H 1.113668 3.176408 1.519466
H 0.291226 3.233444 -0.084472
H -0.726116 -0.160827 2.319830
H -0.291237 -1.404768 3.130862
O -2.370052 0.511741 1.869981
H -2.399977 1.415332 1.533658
H -2.527457 -0.023387 1.066861

MISO.3H2O

E= -967.643776
N 0.521485 -1.367394 -0.866316
C -0.027576 -2.485647 -0.304525
C -1.388912 -2.257366 -0.210042
N -1.682060 -1.014820 -0.679314
C -0.521208 -0.505687 -1.056276
N -0.390879 0.808923 -1.614870
O -1.279160 1.621871 -1.355095
C 1.969207 -1.175548 -1.008567
C 2.619922 -0.514959 0.228918
C 2.445913 1.000524 0.295623
O 1.108730 1.332480 0.624505
C 0.919244 2.741840 0.679788
O 0.589134 1.049247 -2.322587
O 2.256123 -1.177218 1.414336
O -0.256768 -0.761483 2.423243
H 0.595623 -3.313330 0.008915
H -2.153831 -2.917677 0.182761
H 2.387332 -2.182160 -1.116796
H 2.163491 -0.598034 -1.914456
H 3.701413 -0.678813 0.091219
H 1.343322 -0.920399 1.692696
H 2.722313 1.442563 -0.680011
H 3.124886 1.399200 1.070546
H -0.080758 2.923937 1.086316
H 1.670719 3.205611 1.343182
H 1.003525 3.180778 -0.329480
H -0.802777 -0.008312 2.085470
H -0.111038 -0.558931 3.353777
O -1.880679 1.262983 1.560286
H -1.626535 1.500851 0.655263
H -2.777803 0.898846 1.427290
O -4.081216 0.131889 0.296681
H -3.349313 -0.280039 -0.213652
H -4.699246 -0.578151 0.499983

MISO.4H2O
E= -1044.078743
C 0.676753 -1.257035 0.490094
N -0.320960 -1.867839 -0.216130
C 0.255963 -2.270619 -1.387026
C 1.590600 -1.909005 -1.320350
N 1.837841 -1.272290 -0.144891
C -1.764587 -1.955814 0.035184
C -2.542887 -0.697269 -0.415468
O -2.225606 -0.340216 -1.735637
N 0.507440 -0.682331 1.791040
O -0.438669 -1.065637 2.482492
O 1.327753 0.166673 2.145691
C -2.498409 0.466829 0.574152
O -1.226246 1.106699 0.565468
C -1.113889 2.049670 1.636563
O 0.218836 0.755365 -2.259277
O 1.722276 1.933652 -0.205343
O 4.062461 0.497201 -0.065076
H -0.333981 -2.732704 -2.168238
H 2.364113 -2.062698 -2.064163
H -2.112915 -2.801357 -0.567405
H -1.928338 -2.167758 1.093511
H -3.597551 -1.018696 -0.421286
H -1.313014 0.036301 -1.795283
H -2.716504 0.083765 1.586706
H -3.274635 1.200474 0.296543
H -0.170695 2.586664 1.496917
H -1.947655 2.770276 1.601429
H -1.112525 1.521833 2.603328
H 0.777959 1.031719 -1.500787
H -0.114709 1.610462 -2.573437
H 1.521373 1.489567 0.633130
H 2.661368 1.693741 -0.341847
H 3.414364 -0.236940 0.012170
H 4.721347 0.210009 -0.706117
H 0.244968 3.314613 -0.981450
O -0.668043 3.334121 -1.307240
H -1.056583 2.564536 -0.861381

MISO.5H2O
E= -1120.518001
N 1.189514 1.928584 -0.695150
C 1.559591 0.888985 0.036651
N 2.053303 -0.137885 -0.717051
C 1.957899 0.281212 -2.012987
C 1.438314 1.563552 -1.980923
N 1.465577 0.866294 1.465674
O 0.650907 1.630459 1.992223
C 2.428849 -1.508549 -0.350624
C 1.220756 -2.473978 -0.299154
C 0.406876 -2.387609 0.989704
O -0.322950 -1.174519 1.054865
C -1.049476 -1.079733 2.275494
O 0.450042 -2.396633 -1.468848
O 2.199657 0.100575 2.090367
O -1.159874 -0.252969 -1.767249
O -1.772431 1.560298 0.412484
O -0.754677 3.951950 -0.407044
O -3.948563 -0.014175 0.777331
H 2.221377 -0.376562 -2.831058
H 1.221197 2.219037 -2.816588
H 3.102475 -1.848182 -1.144689
H 2.963817 -1.487681 0.600603
H 1.675991 -3.478517 -0.281424
H -0.117357 -1.580122 -1.470995
H 1.092690 -2.465011 1.854736
H -0.294163 -3.240791 1.019191

H -1.677379 -0.186697 2.222512
H -1.699178 -1.961912 2.407358
H -0.354503 -1.002833 3.129588
H -1.252340 0.325793 -0.988474
H -2.040130 -0.682515 -1.834499
H -1.036494 1.536304 1.046796
H -1.695381 2.457607 0.023695
H 0.058107 3.399535 -0.465007
H -0.842309 4.388709 -1.261218
H -3.210617 0.632835 0.687454
H -4.749412 0.512669 0.872619
O -3.677103 -1.421130 -1.563775
H -3.558210 -2.354337 -1.353844
H -3.863860 -0.980406 -0.702863

[MISO]-
E= -738.384616
N -3.180984 -0.627991 -0.360124
C -2.088947 0.031570 0.043951
N -1.053605 -0.841729 0.355148
C -1.537234 -2.116873 0.102904
C -2.835157 -1.952427 -0.325500
N -1.984527 1.412497 0.136771
O -0.785649 1.936371 0.351577
C 0.283127 -0.591008 0.869641
C 1.329994 -0.361444 -0.241460
O 0.972222 0.668174 -1.127877
O -3.016696 2.142487 -0.015872
C 2.671518 -0.080347 0.435621
O 3.734872 -0.256614 -0.494530
C 4.971713 0.147855 0.044340
H -0.915544 -2.992385 0.256324
H -3.540117 -2.728502 -0.614710
H 0.242723 0.283989 1.524680
H 0.575862 -1.475903 1.454131
H 1.432788 -1.294101 -0.824166
H 0.312691 1.260506 -0.653341
H 2.662180 0.960176 0.808868
H 2.829783 -0.763633 1.296869
H 5.739363 -0.019637 -0.724036
H 4.956956 1.220680 0.318559
H 5.229053 -0.437934 0.950871

[MISO]-.H2O
E= -814.829315
C 2.303779 -2.751173 0.088678
N 2.833234 -1.513705 0.341929
C 1.865619 -0.648719 0.027869
N 0.730916 -1.295896 -0.438417
C 1.014913 -2.650884 -0.384411
N 1.970514 0.729998 0.152350
O 3.094007 1.269749 0.419959
C -0.535945 -0.768459 -0.921482
C -1.580927 -0.576750 0.199092
C -2.814666 0.074598 -0.425307
O -3.939052 -0.095339 0.429087
C -5.056527 0.632719 -0.027312
O 0.860221 1.457153 0.033696
O -1.091511 0.168937 1.286647
H 0.280584 -3.388926 -0.688233
H 2.877893 -3.657918 0.262520
H -0.345135 0.186151 -1.419396
H -0.929359 -1.489094 -1.652919
H -1.874569 -1.568921 0.582221
H -0.369459 0.763645 0.940624
H -2.604276 1.150016 -0.570647
H -3.039980 -0.376541 -1.414802
H -5.880057 0.451230 0.677063
H -4.838448 1.717431 -0.063890
H -5.364654 0.305746 -1.041513
H 2.884864 3.522575 -0.236629
O 2.054905 3.936498 -0.514248
H 1.453917 3.177849 -0.349321

[MISO]-.2H2O
E= -891.274472
C 1.959895 -2.230249 -0.105719
C 2.589332 -1.772344 -1.239792
N 2.495702 -0.410027 -1.335415
C 1.805062 -0.031171 -0.257704
N 1.459099 -1.110528 0.543292
N 1.484092 1.283024 0.043462
O 0.963592 1.555347 1.225056
C 0.556419 -1.217945 1.692464
C -0.941698 -0.972089 1.381706
O -1.329180 0.372345 1.608362
O 1.705940 2.206144 -0.820561
C -1.297356 -1.412874 -0.036028
O -2.711828 -1.506515 -0.132810
C -3.150656 -1.492790 -1.482106
O -0.571101 3.646151 -0.284257
O -2.509055 1.589528 -0.796658
H 1.813357 -3.223007 0.304613
H 3.112905 -2.357492 -1.991462
H 0.675213 -2.246586 2.057910
H 0.872631 -0.518972 2.472214
H -1.525117 -1.591384 2.082917
H -0.520552 0.934476 1.434881
H -0.912007 -0.664500 -0.747800
H -0.834869 -2.390142 -0.273664
H -4.245374 -1.584076 -1.464595
H -2.866564 -0.543363 -1.964278
H -2.722580 -2.345688 -2.046438
H -2.342738 1.091265 0.021926

H -1.885332 2.340884 -0.730991
H 0.257372 3.265730 -0.661204
H -0.461527 3.368359 0.636617

[MISO]-.3H2O
E= -967.719683
N -0.518923 1.419961 -0.787781
C -0.138480 2.598025 -0.168111
C 1.209622 2.490010 0.078311
N 1.678740 1.276766 -0.360340
C 0.619023 0.644541 -0.879547
N 0.659177 -0.612976 -1.456512
O 1.634014 -1.412665 -1.122069
C -1.907658 1.101140 -1.092937
C -2.664069 0.325217 0.012165
C -2.402970 -1.181370 0.010330
O -1.164726 -1.469218 0.621580
C -0.785120 -2.822654 0.425153
O -0.282068 -0.975969 -2.261051
O -2.514961 0.917289 1.284104
O -0.021050 0.629406 2.448659
H -0.866464 3.368270 0.053985
H 1.869305 3.219384 0.538898
H -2.412458 2.068227 -1.223926
H -1.931884 0.531955 -2.026517
H -3.729269 0.433978 -0.260585
H -1.627930 0.689778 1.658279
H -2.405837 -1.540503 -1.034048
H -3.215043 -1.686199 0.572384
H 0.113463 -2.994985 1.026423
H -1.594318 -3.501740 0.762771
H -0.562272 -2.999658 -0.639600
H 0.518962 -0.142079 2.149935
H 0.392914 1.376158 1.995122
O 1.784152 -1.325788 1.610767
H 1.635076 -1.433011 0.637068
H 2.635952 -0.858424 1.602249
O 4.036395 -0.074840 0.222181
H 3.831210 -0.849939 -0.319485
H 3.259509 0.497360 -0.006074

[MISO]-.4H2O
E= -1044.158311
C 0.382107 -0.591308 1.201540
N -0.761303 -1.329381 0.952426
C -0.338715 -2.538930 0.426239
C 1.032943 -2.478528 0.368921
N 1.481383 -1.269969 0.843272
C -2.170156 -0.968561 1.064593
C -2.771348 -0.220569 -0.145153
O -2.645277 -0.931606 -1.358524
N 0.384796 0.655400 1.797639
O -0.685776 1.076072 2.389534
O 1.465883 1.368976 1.747359
C -2.388963 1.256668 -0.229441
O -1.039008 1.449512 -0.622155
C -0.582530 2.766000 -0.293736
O -0.166828 -1.442743 -2.512753
O 2.638012 1.638437 -0.672715
O 4.007644 -0.825929 -0.148745
H -1.053594 -3.293717 0.123326
H 1.725409 -3.241422 0.025112
H -2.716408 -1.916311 1.165171
H -2.292487 -0.360116 1.964135
H -3.854797 -0.200912 0.071413
H -1.714039 -0.972049 -1.677017
H -2.540247 1.698080 0.769500
H -3.056050 1.755767 -0.958175
H 0.445703 2.849952 -0.658860
H -1.220000 3.522266 -0.790223
H -0.600186 2.900250 0.797211
H 0.246258 -1.853166 -1.738995
H 0.339082 -0.604267 -2.648255
H 2.165884 1.562845 0.199182
H 3.309456 0.936693 -0.597450
H 3.146785 -0.914146 0.347603
H 3.819913 -1.246473 -0.995971
H 1.564264 1.245954 -1.985395
O 0.865975 1.059997 -2.665350
H 0.045903 1.248056 -2.176670

[MISO]-.5H2O
E= -1120.596621
N 1.078706 2.148000 -0.085496
C 1.320045 0.992121 0.546665
N 2.176167 0.196131 -0.184307
C 2.473559 0.899567 -1.340314
C 1.795512 2.086560 -1.255286
N 0.807298 0.642564 1.783098
O -0.267077 1.254422 2.203706
C 2.582260 -1.186937 0.018197
C 1.564359 -2.237231 -0.487857
C 0.439253 -2.554278 0.501867
O -0.576194 -1.562390 0.456506
C -1.412501 -1.602997 1.612489
O 1.107283 -1.956048 -1.791413
O 1.369797 -0.296613 2.468885
O -0.719891 0.120752 -2.099781
O -1.972314 1.376570 0.205710
O -0.965016 3.927570 -0.416041
O -4.207804 -0.016913 -0.107369
H 3.090359 0.460688 -2.113844
H 1.771533 2.898209 -1.975836
H 3.505532 -1.314488 -0.563123

H 2.780942 -1.338962 1.082428
H 2.145262 -3.173768 -0.560098
H 0.523500 -1.158481 -1.787189
H 0.866184 -2.599257 1.516976
H -0.004883 -3.534518 0.243570
H -2.185546 -0.843932 1.463151
H -1.880500 -2.601378 1.707520
H -0.821360 -1.358821 2.506237
H -0.954063 0.584421 -1.270830
H -1.460928 -0.506262 -2.197637
H -1.349757 1.301440 0.994197
H -1.902026 2.319240 -0.036960
H -0.188509 3.361320 -0.137025
H -0.943886 3.873808 -1.378805
H -3.412009 0.563466 0.048585
H -4.491993 -0.285038 0.772697
O -2.636096 -1.928034 -1.490829
H -1.880632 -1.949123 -0.873804
H -3.278836 -1.340802 -1.042843

[MISO-NO2], I
E= -533.150187
C -2.067521 0.930154 -0.299259
C -3.435572 0.823589 -0.366353
N -3.845026 -0.465713 -0.069667
C -2.741908 -1.131095 0.176740
N -1.611459 -0.336216 0.051505
C -0.309117 -0.752152 0.221964
C 0.824863 0.160311 -0.073793
O 0.832192 1.241558 0.879187
C 2.143309 -0.603255 -0.035923
O 3.177575 0.322337 -0.326499
C 4.467796 -0.259435 -0.218099
H -1.383423 1.761330 -0.406983
H -4.151969 1.604194 -0.602913
H -0.170810 -1.761272 0.599893
H 0.704144 0.589010 -1.086844
H 1.643980 1.744298 0.719803
H 2.290681 -1.037480 0.970572
H 2.131411 -1.418538 -0.782945
H 5.198432 0.525452 -0.448189
H 4.642665 -0.639889 0.804654
H 4.584517 -1.092850 -0.934634
H -2.663457 -2.180614 0.441544

[MISO-NO2], II
E= -533.128149
C -2.314627 0.945712 0.301990
C -3.514943 0.618291 -0.290257
N -3.555865 -0.728602 -0.591685
C -2.396756 -1.205955 -0.193318
N -1.604107 -0.234036 0.366704
C -0.262484 -0.408849 0.855192
C 0.830755 0.149844 -0.132724
O 0.681402 1.505165 -0.124177
C 2.204461 -0.301600 0.370804
O 3.162954 0.078607 -0.594223
C 4.479850 -0.274579 -0.202285
H -1.896585 1.879415 0.658843
H -4.348662 1.276450 -0.514810
H -0.072910 -1.483308 0.970551
H -0.142003 0.090957 1.823865
H 0.622356 -0.286252 -1.128272
H 2.413379 0.182100 1.343665
H 2.213499 -1.401930 0.507024
H 5.153323 0.049125 -1.004997
H 4.759166 0.230081 0.740744
H 4.570868 -1.368224 -0.062610
H -2.065005 -2.237313 -0.268343

[MISO-NO2], III
E= -533.112165
C -3.535266 0.666598 -0.275724
N -3.660315 -0.693213 -0.576747
C -2.542305 -1.209725 -0.229660
N -1.649637 -0.328969 0.299458
C -2.295336 0.908451 0.267147
C -0.303542 -0.611355 0.769521
C 0.775368 0.041457 -0.088950
C 2.146118 -0.529629 0.251322
O 3.100466 0.164059 -0.536269
C 4.433432 -0.216045 -0.229870
O 0.734356 1.444575 0.165634
H -1.796748 1.809238 0.603888
H -4.340215 1.367659 -0.471620
H -0.179103 -1.700968 0.755939
H -0.196130 -0.253437 1.803261
H 0.561459 -0.156299 -1.152720
H 1.483892 1.841851 -0.297397
H 2.359563 -0.381009 1.326997
H 2.174101 -1.612367 0.026747
H 5.095905 0.373704 -0.875033
H 4.667478 -0.008478 0.830062
H 4.590083 -1.292204 -0.426737

[MISO-OH], I
E= -662.523775
C -1.236328 -0.051580 -0.571691
N -0.118110 -1.029668 -0.415572
C 1.163488 -0.571014 -0.107585
N 1.407185 0.777456 -0.000209
C 0.419437 1.817804 -0.241845
C -0.932948 1.258152 0.179425
C 2.747379 0.902788 0.302250

C 3.233396 -0.388706 0.361614
N 2.241248 -1.303351 0.107175
O -0.331972 -2.252065 -0.711403
O -1.919269 2.225587 -0.127926
O -2.466255 -0.593739 -0.183725
C -2.523156 -1.166004 1.132918
H 3.211262 1.872099 0.444674
H 4.248018 -0.705496 0.582494
H 0.392395 2.096234 -1.306151
H 0.670742 2.704275 0.351203
H -1.346957 0.170427 -1.643553
H -1.834746 -2.017488 1.211949
H -2.295524 -0.419664 1.910481
H -3.555923 -1.510061 1.255242
H -2.784556 1.799449 -0.053885
H -0.904605 1.052257 1.261708

[MISO-OH], II
E= -662.517746
C -1.392428 0.583277 0.036073
N -1.612888 -0.744250 -0.186408
C -2.911883 -0.856891 -0.632654
C -3.380078 0.448552 -0.669463
N -2.422866 1.352214 -0.249464
C -0.518781 -1.597349 0.252637
C 0.613412 -0.569322 0.524605
O 1.190352 -0.811002 1.767623
N -0.112709 0.757496 0.532189
O 0.408410 1.814659 0.999995
C 1.647826 -0.588770 -0.606537
O 2.696879 0.289068 -0.281916
C 3.691412 0.338068 -1.294009
H -3.371210 -1.804397 -0.888188
H -4.363228 0.783058 -0.986595
H -0.765846 -2.125217 1.183624
H 2.022707 -1.626029 -0.706095
H 1.155211 -0.296671 -1.553008
H 4.464174 1.035702 -0.950683
H 4.137704 -0.660464 -1.455980
H 3.263196 0.700811 -2.245530
H -0.235279 -2.321468 -0.521760
H 1.869805 -0.130736 1.900456

[MISO-OH], III
E= -662.517623
O -0.160430 1.844187 -0.104578
N 1.104021 1.669142 -0.669260
C 1.702913 0.524197 -0.301683
N 1.170430 -0.408161 0.571770
C -0.108068 -0.217843 1.227782
C -0.933620 0.673108 0.305368
C 2.086511 -1.417420 0.691302
C 3.151007 -1.052120 -0.135495
N 2.919200 0.133162 -0.745158
C -1.383101 -0.041186 -0.985080
O -1.956037 -1.312413 -0.756669
C -3.309757 -1.282496 -0.307633
H 1.915471 -2.282251 1.323022
H 4.065878 -1.612299 -0.307704
H 0.011461 0.284239 2.197420
H -0.609243 -1.183431 1.356408
H -2.085336 0.629340 -1.513963
H -0.507279 -0.200457 -1.624771
H -3.389684 -0.896952 0.719955
H -3.922577 -0.652811 -0.977917
H -3.674488 -2.316201 -0.348693
O -1.991592 1.169834 1.062331
H -2.530602 1.741518 0.498747

[MISO-OH], IV
E= -662.465824
N -1.258108 -0.846906 0.053846
C -2.202880 -1.856580 0.051851
C -3.431053 -1.246602 -0.143100
N -3.299677 0.098872 -0.256302
C -1.997827 0.346957 -0.132922
N -1.333372 1.564960 -0.076325
O -2.017775 2.565596 -0.291848
C 0.086980 -1.066276 0.158456
C 1.130152 0.006820 0.082797
C 2.507965 -0.638096 0.013464
O 3.442240 0.331839 -0.400737
C 4.769898 -0.164194 -0.375620
O 1.083166 0.853294 1.228465
H -1.912641 -2.894164 0.169674
H -4.401677 -1.731808 -0.199688
H 0.354135 -2.103258 0.354674
H 0.976306 0.604857 -0.831250
H 0.358738 1.478551 1.050597
H 2.770187 -1.035614 1.012827
H 2.487802 -1.481934 -0.705444
H 5.424826 0.649500 -0.709591
H 5.056002 -0.471595 0.647098
H 4.883966 -1.031018 -1.054031

[MISO-OH], V
E= -662.453010
C 0.134377 1.387197 0.706474
C -0.883785 2.275941 0.430984
N -1.999243 1.623717 -0.003387
C -1.676862 0.345964 0.000193
N -0.361625 0.119035 0.434177
N -2.592440 -0.685728 -0.395975
O -2.186591 -1.861239 -0.397332

C 0.317682 -1.065034 0.604819
C 1.681034 -1.111184 1.205018
H 1.808857 -0.335443 1.975088
O -3.725698 -0.345039 -0.713886
C 2.821649 -0.979259 0.184632
O 2.811849 0.335719 -0.342487
C 3.796962 0.517480 -1.349248
H 1.162555 1.530392 1.005402
H -0.858207 3.357703 0.518220
H -0.209827 -1.950310 0.279117
H 1.796530 -2.088939 1.695300
H 2.691513 -1.716207 -0.629307
H 3.787776 -1.181926 0.684361
H 3.717386 1.554317 -1.697769
H 4.810435 0.339649 -0.945539
H 3.623310 -0.170565 -2.196175

[MISO-CH2NO2]-
E= -493.963249
C 2.248159 1.111138 -0.037918
N 1.124063 0.338753 0.152171
C 1.522932 -0.964189 0.091211
N 2.829656 -1.073126 -0.133080
C 3.290166 0.222280 -0.214416
C -0.232114 0.831649 0.420632
C -1.327569 -0.017006 -0.257004
C -2.688368 0.755629 -0.112088
O -3.714779 -0.214781 -0.203669
O -1.462858 -1.261313 0.270153
H -2.817383 1.518899 -0.901266
H -2.723437 1.263281 0.878688
H -0.265178 1.875164 0.064934
H -0.416744 0.811839 1.506207
H -1.070909 -0.022815 -1.355338
H -3.163481 -1.022068 0.032921
H 2.198720 2.194599 -0.036860
H 4.337360 0.448549 -0.400855
H 0.786875 -1.755072 0.203562

[MISO-CH2NO2]-.H2O
E= -570.414407
C -3.689498 0.042793 -0.388155
C -2.769680 -0.962100 -0.166062
N -1.599833 -0.328775 0.192960
C -1.855659 1.011305 0.179451
N -3.111247 1.273478 -0.170355
C -0.344881 -0.992580 0.561476
C 0.896882 -0.362385 -0.107081
O 1.105616 0.911297 0.366088
C 2.084446 -1.320717 0.148858
O 3.250626 -0.996424 -0.593926
H 1.809566 -2.350805 -0.143259
H 2.294558 -1.306001 1.238395
H -0.459628 -2.048073 0.272174
H -0.211917 -0.927895 1.652531
H 0.709248 -0.394808 -1.211945
H 3.466594 -0.051781 -0.405879
H -2.831200 -2.042868 -0.232400
H -4.727347 -0.057245 -0.696532
H -1.062587 1.715058 0.407371
H 2.388671 1.400814 0.177683
O 3.427029 1.682915 0.007353
H 3.405776 2.170485 -0.823400

[MISO-CH2NO2]-.2H2O
E= -646.858069
C 3.858593 0.258126 0.303414
C 3.092362 -0.888056 0.342791
N 1.803793 -0.488304 0.061346
C 1.843040 0.862199 -0.139865
N 3.071160 1.346854 0.003371
C 0.654256 -1.392699 -0.045304
C -0.701506 -0.678511 -0.015049
O -0.967184 0.024360 -1.173862
C -1.815376 -1.756851 0.184590
O -3.013318 -1.229252 -0.369575
H -1.974423 -1.983531 1.250690
H -1.535831 -2.692769 -0.343024
H 0.727154 -2.108949 0.790390
H 0.719634 -1.945634 -0.995002
H -0.715820 -0.016694 0.879650
H -2.609839 -0.587489 -1.020578
H 3.332899 -1.926271 0.545098
H 4.927037 0.354628 0.480220
H 0.950243 1.426331 -0.386707
O -3.429228 1.175866 1.248105
H -2.734759 1.679924 0.774798
H -3.428440 0.318635 0.785648
O -1.382790 2.372312 -0.352437
H -1.216166 1.426094 -0.764511
H -1.814399 2.874338 -1.051012

[MISO-CH2NO2]-.3H2O
E= -723.300573
C 3.065832 -0.949315 0.923574
C 1.955268 -1.762875 1.010010
N 1.146463 -1.408386 -0.049186
C 1.783995 -0.406846 -0.715969
N 2.950312 -0.105270 -0.158873
C -0.209665 -1.887214 -0.314016
C -1.240934 -0.761901 -0.121023
O -1.095273 0.238665 -1.056092
C -2.679049 -1.338071 -0.223087
O -3.546417 -0.248240 -0.516278

H -3.002203 -1.810185 0.718292
H -2.724905 -2.090244 -1.037833
H -0.396608 -2.731183 0.367441
H -0.271314 -2.239301 -1.353454
H -1.109926 -0.379345 0.918298
H -2.914036 0.334422 -1.013215
H 1.667042 -2.540434 1.708374
H 3.932824 -0.926834 1.577697
H 1.318194 0.092316 -1.556152
O -2.759067 1.800071 1.553741
H -1.919152 2.051209 1.122920
H -3.131878 1.128909 0.957635
O -0.148338 2.171388 0.259003
H -0.514618 1.399798 -0.346804
H 0.327394 1.716892 0.964154
O 2.495935 2.851900 -0.726977
H 2.880556 1.978297 -0.544933
H 1.553805 2.738317 -0.490124

[MISO-CH2NO2]-.4H2O
E= -799.748423
C -3.311984 -0.138251 -0.333919
C -2.251922 -0.187653 0.551839
N -1.497275 -1.268055 0.156724
C -2.118599 -1.809969 -0.931281
N -3.222326 -1.159377 -1.258052
C -0.238262 -1.716417 0.740506
C 0.985599 -0.916596 0.267708
O 1.056911 0.353058 0.817374
C 2.264218 -1.703033 0.667454
O 3.365332 -0.799288 0.659780
H 2.474070 -2.518902 -0.040825
H 2.137528 -2.130547 1.682558
H -0.120324 -2.779123 0.476159
H -0.310279 -1.628087 1.832872
H 0.949326 -0.870595 -0.840888
H 2.909290 0.038475 0.911899
H -1.948095 0.444644 1.380897
H -4.133222 0.572865 -0.348808
H -1.716157 -2.680667 -1.439806
O 3.256894 0.274606 -2.070163
H 2.524168 0.887248 -1.867615
H 3.431436 -0.153363 -1.213439
O 1.200208 2.036749 -1.017182
H 1.144755 1.341269 -0.219800
H 1.766435 2.743795 -0.688691
O -0.579507 2.050496 2.208713
H -0.910750 2.508475 1.416201
H 0.028944 1.378026 1.820339
O -1.400582 2.841570 -0.536058
H -1.878719 2.005247 -0.624204
H -0.479555 2.607249 -0.801104

[MISO-CH2NO2]-.5H2O
E= -876.186023
C -3.116688 -1.493879 0.080270
C -1.989213 -2.172695 0.499962
N -0.950541 -1.685248 -0.256409
C -1.474389 -0.741172 -1.081526
N -2.782483 -0.600298 -0.912866
C 0.467633 -2.030429 -0.125400
C 1.316594 -0.819519 0.297538
O 1.001531 -0.355523 1.559435
C 2.805644 -1.208441 0.203231
O 3.276020 -1.287095 -1.144050
H 2.973688 -2.192766 0.670499
H 3.369923 -0.455497 0.776058
H 0.833081 -2.427804 -1.082959
H 0.541005 -2.804447 0.649735
H 1.160542 -0.044519 -0.487675
H 3.303263 -0.355079 -1.438815
H -1.825250 -2.922212 1.264864
H -4.136570 -1.597415 0.438296
H -0.861146 -0.163334 -1.762927
O 3.241551 1.527881 -1.281497
H 2.698628 1.735190 -0.464224
H 4.109068 1.897378 -1.087043
O 1.853718 2.070373 0.940178
H 1.571851 1.178607 1.325327
H 1.008029 2.483971 0.687543
O -0.896618 2.842912 0.368623
H -1.084511 2.038163 0.952620
H -1.182008 3.596616 0.896090
O -1.234745 0.750047 1.888001
H -0.330943 0.227177 1.753004
H -1.928306 0.161629 1.566126
O -3.073989 2.277185 -1.506933
H -3.166833 1.316406 -1.367878
H -2.319567 2.517329 -0.938234

[MISO-C2H4O2]-, I
E= -509.343171
C -0.736439 2.293453 0.141085
N 0.545380 1.823104 0.233589
C 0.462654 0.512529 -0.049570
N -0.848368 0.139247 -0.342764
C -1.615587 1.289110 -0.198128
N 1.531773 -0.369627 -0.072700
C -1.437837 -1.186033 -0.497568
C -1.520651 -1.936106 0.834630
H -2.027298 -2.905187 0.691901
H -0.499373 -2.117735 1.193547
H -2.080676 -1.347221 1.577766
H -2.687974 1.271974 -0.362904

H -0.967340 3.342848 0.314294
H -0.828426 -1.757812 -1.201539
H -2.444438 -1.025127 -0.914982
O 2.689885 0.038386 0.294203
O 1.312764 -1.594451 -0.470158

[MISO-C2H4O2]-, II
E= -509.414029
N 2.578324 0.587538 -0.128590
C 1.234917 0.798763 0.120205
N 0.577851 -0.461214 0.164423
C 1.514459 -1.413484 -0.073389
C 2.731160 -0.740000 -0.252756
C -0.805680 -0.715806 0.566287
C -1.869560 -0.114226 -0.404681
O -1.989488 1.193250 -0.336153
N 0.665507 1.964732 0.270705
H -1.654466 -0.530593 -1.428298
O -3.127066 -0.823556 0.012794
H 1.266535 -2.470384 -0.080776
H 3.697415 -1.192767 -0.470283
H -0.983382 -0.260981 1.552006
H -0.922135 -1.806053 0.632992
H -3.684060 -0.062065 0.224951
H -0.371026 1.836409 0.276517

[MISO-C2H4O2]-.H2O
E= -585.790757
C 2.598677 -0.231488 -0.193308
C 2.403179 -1.553929 0.136524
N 1.069441 -1.849681 0.225484
C 0.436121 -0.701493 -0.049542
N 1.333483 0.322792 -0.332052
N -0.940376 -0.542148 -0.071004
O -1.422707 0.614717 -0.469241
C 1.113294 1.757979 -0.492859
C 0.766770 2.436844 0.834157
O -1.698077 -1.511974 0.288635
H 0.667340 3.524371 0.685640
H -0.191153 2.039196 1.192574
H 1.552726 2.249886 1.582084
H 3.491027 0.364431 -0.353181
H 3.163117 -2.313695 0.305705
H 0.301717 1.908808 -1.208403
H 2.050775 2.163614 -0.903072
H -3.893028 -0.692824 0.261343
O -4.136266 0.205494 -0.006608
H -3.232213 0.526090 -0.231806

[MISO-C2H4O2]-.2H2O
E= -662.236729
C 0.084102 2.577255 0.097722
C 1.380762 2.231628 0.399082
N 1.579024 0.888789 0.215230
C 0.400933 0.407021 -0.200717
N -0.547340 1.409710 -0.304642
N 0.144546 -0.918828 -0.513451
O -0.944779 -1.196677 -1.184821
C -1.998124 1.293003 -0.456469
C -2.646530 0.642565 0.767444
O 0.939608 -1.842836 -0.097570
H -3.740689 0.624483 0.641795
H -2.291733 -0.390851 0.875362
H -2.404298 1.210338 1.678865
H -0.447776 3.521908 0.120322
H 2.186995 2.882461 0.726443
H -2.205984 0.699290 -1.349949
H -2.368632 2.319421 -0.596628
H -0.816154 -3.069205 0.977351
O -1.755144 -2.978601 0.757380
H -1.673198 -2.434603 -0.059162
H 3.075314 -1.666254 0.043418
O 3.755617 -0.988529 0.183415
H 3.163271 -0.210366 0.252623

[MISO-C2H4O2]-.3H2O
E= -738.678148
C 0.842297 -2.548300 -0.204112
C -0.497481 -2.510539 0.097877
N -0.939100 -1.215009 0.166959
C 0.132950 -0.453408 -0.091993
N 1.251295 -1.229559 -0.341614
N 0.122346 0.929071 -0.125438
O 1.231392 1.558614 -0.389005
C 2.650014 -0.845844 -0.532767
C 3.322934 -0.460668 0.785530
O -0.978985 1.558431 0.125675
H 4.386922 -0.236307 0.610328
H 2.832903 0.435497 1.186080
H 3.248515 -1.282868 1.513331
H 1.539951 -3.366452 -0.342757
H -1.173599 -3.343229 0.268569
H 2.687024 -0.007390 -1.231677
H 3.142873 -1.720912 -0.981187
H -0.363824 3.853892 0.551059
O 0.544544 4.162923 0.428323
H 0.944401 3.331300 0.094418
H -2.374245 0.991961 -0.835226
O -3.095027 0.532090 -1.327922
H -2.833278 -0.391327 -1.214941
H -2.650827 -1.003346 0.983940
O -3.601954 -0.994193 1.226893
H -3.932660 -0.282720 0.659789

[MISO-C2H4O2]-.4H2O
E= -815.122657
C -2.445604 -0.371959 -1.097920
C -2.055577 0.924005 -1.346208
N -0.711219 1.069426 -1.118073
C -0.281134 -0.137816 -0.733713
N -1.305090 -1.060297 -0.720187
N 1.027329 -0.441447 -0.397950
O 1.400107 -1.684052 -0.395653
C -1.327655 -2.392001 -0.105754
C -1.111462 -2.320707 1.406982
O 1.841153 0.529132 -0.097469
H -1.271011 -3.318060 1.845851
H -0.079610 -2.009905 1.610738
H -1.796769 -1.595965 1.867347
H -3.407339 -0.868649 -1.150911
H -2.665865 1.756281 -1.683289
H -0.551494 -3.002987 -0.575245
H -2.317182 -2.808465 -0.341834
H 4.024065 -0.446352 0.111712
O 4.180316 -1.395802 0.014346
H 3.256333 -1.687482 -0.134195
H 1.686404 2.906511 -0.665214
O 0.835973 3.359684 -0.746017
H 0.248115 2.591896 -0.959728
H -2.234513 0.950970 1.119872
O -1.983854 0.982182 2.051940
H -1.072651 1.357605 2.031457
O 0.584301 2.057854 1.840981
H 0.478806 2.783638 1.200639
H 1.090176 1.416081 1.290920

[MISO-C2H4O2]-.5H2O
E= -891.565442
C 1.968334 -2.331947 -0.407132
C 0.617282 -2.576773 -0.415421
N -0.083392 -1.400086 -0.338795
C 0.842666 -0.433437 -0.278921
N 2.119840 -0.956350 -0.332669
N 0.564529 0.919176 -0.192439
O 1.540382 1.767008 -0.171241
C 3.420892 -0.305050 -0.163389
C 3.701586 0.035263 1.300502
O -0.679063 1.312642 -0.144747
H 4.708947 0.469326 1.394711
H 2.965975 0.771890 1.646388
H 3.644156 -0.868004 1.926070
H 2.830938 -2.986699 -0.450328
H 0.103510 -3.531796 -0.464745
H 3.437268 0.601159 -0.772565
H 4.163666 -1.018253 -0.548103
H -0.666173 3.833451 -0.199261
O 0.197131 4.264492 -0.247190
H 0.780898 3.478847 -0.221244
H -1.694005 0.608439 -1.408932
O -2.239183 0.011902 -1.976663
H -1.834646 -0.840697 -1.760016
H -1.755163 -1.817251 0.477760
O -2.545498 -2.134372 0.961135
H -3.285877 -1.640634 0.553991
O -4.303912 -0.122444 -0.062411
H -3.704933 -0.018023 -0.832608
H -3.840608 0.364966 0.646915
O -2.384733 0.622378 1.965723
H -1.712877 0.902484 1.306692
H -2.333475 -0.349575 1.941911

[MISO-C2H4O2]
E= -509.299121
C -0.128058 2.403770 0.151728
N 0.982174 1.622775 0.232589
C 0.557146 0.400514 -0.025925
N -0.791736 0.340328 -0.284953
C -1.234144 1.628845 -0.165363
N 1.434051 -0.747037 -0.050137
C -1.682776 -0.802925 -0.550308
C -2.041865 -1.550034 0.732054
H -2.745429 -2.360738 0.495286
H -1.141884 -1.988044 1.180355
H -2.517266 -0.870667 1.453876
H -2.278890 1.877223 -0.317118
H -0.086888 3.475578 0.318460
H -1.188018 -1.464241 -1.265317
H -2.578134 -0.375363 -1.018474
O 2.623462 -0.560128 0.158660
O 0.919446 -1.850776 -0.281495

CH2NO2
E= -244.353158
O 0.671863 1.100530 -0.000117
N 0.101737 0.000000 0.000015
O 0.671863 -1.100530 -0.000117
C -1.311011 0.000000 0.000219
H -1.797952 0.971051 0.000222
H -1.797952 -0.971051 0.000225

CH3OH
E= -115.693603
C -0.669529 -0.020129 0.000000
O 0.751662 0.122620 0.000000
H -1.086680 0.995366 -0.000000
H -1.028535 -0.549800 0.899742
H -1.028535 -0.549801 -0.899742
H 1.147625 -0.755949 -0.000000

CH4
E= -40.477883
C -0.000000 -0.000000 0.000000
H 0.897278 -0.000000 -0.634470
H -0.897278 0.000000 -0.634470
H -0.000000 0.897278 0.634470
H 0.000000 -0.897278 0.634470

CO2
E= -188.602776 in
O 0.000000 0.000000 1.167492
C 0.000000 0.000000 0.000000
O 0.000000 0.000000 -1.167492

CO
E= -113.325302
O 0.000000 0.000000 0.486014
C 0.000000 0.000000 -0.648018

H2O
E= -76.423474
O 0.000000 0.117779 0.000000
H 0.764437 -0.471115 0.000000
H -0.764437 -0.471115 0.000000

NO2-
E= -205.188594
N 0.000000 0.460995 0.000000
O 1.073127 -0.201733 0.000000
O -1.073127 -0.201638 0.000000

NO2-.H2O
E= -281.637966
N -1.296420 -0.077787 -0.000596
O -0.761617 1.059455 0.000290
O -0.510797 -1.072987 0.000201
H 1.598434 0.892770 -0.000025
O 2.051791 0.036342 -0.000025
H 1.241494 -0.530738 0.000469

NO2-.2H2O
E= -358.080298
O -0.665485 -1.060177 -0.111231
N -1.117363 0.127994 0.007433
O -2.349762 0.246815 0.074795
O 1.319242 1.766202 -0.024344
O 2.004778 -1.084677 -0.031559
H 1.806960 0.930814 -0.129508
H 0.395995 1.436106 0.005494
H 1.001203 -1.084792 -0.110084
H 2.147200 -1.123383 0.920778

NO2-.3H2O
E= -434.523828
N 0.212303 -0.810719 -0.003200
O 0.388478 0.454084 -0.106438
O 1.234999 -1.505416 0.066023
H 3.249247 -0.183760 0.069371
O 3.211073 0.781028 0.006303
H 2.237012 0.882679 -0.057891
H -2.074114 1.901692 0.909566
O -1.987885 1.744754 -0.037358
H -1.103342 1.288776 -0.113785
H -2.769455 -0.112446 -0.106511
O -2.751264 -1.081010 -0.013857
H -1.788673 -1.249435 0.004271

NO2-.4H2O
E= -510.965877
O -0.657662 1.443532 0.931842
O 1.034474 0.760945 -1.058614
N 2.203738 0.326096 -0.796072
O 2.353049 -0.196923 0.329633
O -0.951058 -1.434383 -1.045873
O -3.069998 0.364360 -0.363427
H -0.011341 1.164033 0.220888
H -0.201715 2.140340 1.414670
H -0.323174 -0.746069 -1.326437
H -0.672720 -1.583943 -0.117357
H -2.464776 -0.333953 -0.686764
H -2.463291 0.938759 0.129794
O 0.068466 -1.251776 1.589955
H -0.421729 -0.415337 1.670549
H 0.914413 -0.932536 1.199028

NO2-.5H2O
E= -587.408138
O 2.454285 0.016850 0.856652
O 0.134857 -1.283581 1.812685
O -0.860265 1.324567 0.989888
N 0.107366 1.629193 0.225102
O -0.176168 1.777412 -0.980299
O -2.726402 -0.237415 -0.321528
O -0.531802 -1.999568 -0.800654
H 1.916737 0.827781 0.764541
H 1.810982 -0.586326 1.288309
H -0.244857 -0.385788 1.776054
H -0.096068 -1.653728 0.928617
H -1.302616 -1.397461 -0.827672
H 0.189021 -1.508047 -1.252510
H -2.957867 -0.691053 0.497989
H -2.126893 0.486699 -0.010889
O 1.574998 -0.415106 -1.806435

H 1.061635 0.413157 -1.798294
H 2.042354 -0.374867 -0.944331

OH-
E= -75.808437
O 0.000000 0.000000 0.107774
H 0.000000 0.000000 -0.862191

OH-.H2O
E= -152.275900
O -1.243747 -0.098507 -0.050570
H -1.479659 0.673563 0.475836
H -0.141224 -0.044280 -0.069604
O 1.247812 0.088803 -0.071114
H 1.588357 -0.551657 0.567234

OH-.2H2O
E= -228.730202
O -0.030043 1.355293 0.113254
H -0.012552 1.872280 -0.701055
H -0.960180 0.170897 -0.068154
O -1.534217 -0.705970 -0.112201
H -1.735189 -0.872070 0.815385
H 1.125968 0.210288 0.054013
O 1.647858 -0.662958 0.004946
H 0.913175 -1.272324 -0.148185

OH-.3H2O
E= -305.180841
O 0.000481 -0.001390 1.412374
H 0.004199 -0.005498 2.372286
H 0.830415 -0.977354 0.361177
O 1.089771 -1.430788 -0.499721
H 0.226079 -1.447134 -0.936973
H -1.263222 -0.231896 0.362806
O -1.785646 -0.227152 -0.497640
H -1.368262 0.530627 -0.932424
O 0.695109 1.659693 -0.497082
H 0.431629 1.208614 0.363489
H 1.141442 0.919739 -0.933808

OH-.4H2O
E= -381.629611
O -0.002739 -1.502007 -0.524281
O 0.010911 -0.212844 1.848917
O 2.100768 -0.021328 -0.545139
O -2.105053 -0.022167 -0.534521
H -0.004557 -2.455857 -0.638315
H 1.336076 -0.691648 -0.647224
H 2.184050 0.017880 0.416710
H -1.339456 -0.692166 -0.638278
H -2.190328 0.014060 0.427162
H 0.006103 -0.830831 1.065672
H 0.005761 0.656466 1.414167
O -0.002764 1.916696 -0.288776
H 0.766095 1.358304 -0.527926
H -0.772726 1.356998 -0.521565

OH-.5H2O
E= -458.074701
O 0.345829 -0.577898 -1.303110
O -0.233038 -1.425924 1.259945
O -1.313761 1.287623 1.170472
O 0.894327 1.871332 -0.486801
O 2.539384 -0.541058 0.321651
H 0.537808 -0.983873 -2.153336
H 0.003707 -1.329228 0.307317
H -0.562202 -0.530755 1.471064
H -1.823873 0.843865 0.463485
H -0.531819 1.627434 0.681693
H 0.643854 1.028385 -0.970926
H 1.656932 1.556577 0.021480
H 2.037702 -0.864420 1.084333
H 1.843864 -0.595578 -0.387853
H -1.296509 -0.496170 -1.147896
O -2.264931 -0.499131 -0.861915
H -2.251945 -1.175790 -0.171290

Cartesian coordinates of structures optimized at the M06/aug-cc-pVDZ level (in Å) along with zero-point corrected energies (in Hartree)

MISO
E= -737.852191
C 1.973941 -1.765739 -1.029567
N 2.228598 -0.455255 -0.778629
C 1.342316 -0.113579 0.121211
N 0.509936 -1.133457 0.491890
C 0.912148 -2.199687 -0.264774
N 1.256827 1.223018 0.653912
O 1.869708 2.096310 0.078875
C -0.694858 -1.160393 1.319561
C -1.967635 -0.781900 0.560100
C -2.056299 0.671026 0.141897
O -1.128000 0.886531 -0.895425
C -1.147753 2.213938 -1.369390
O 0.565546 1.391769 1.655391
O -2.169727 -1.638372 -0.540865
H 0.402947 -3.155885 -0.193766
H 2.566590 -2.337269 -1.737929
H -0.800588 -2.197967 1.662729
H -0.548514 -0.511802 2.187973
H -2.796291 -0.967814 1.264697
H -1.711759 -1.247197 -1.297109
H -1.840036 1.332048 1.002070
H -3.081096 0.884280 -0.218725
H -0.384015 2.296961 -2.150945
H -2.136753 2.468671 -1.789489
H -0.909389 2.923950 -0.557776

MISO.H2O
E= -814.249763
C -2.065834 -1.237315 -0.316675
C -2.771411 -0.447023 0.566539
N -2.217260 0.794095 0.631462
C -1.209860 0.748380 -0.204020
N -1.063632 -0.460305 -0.825312
N -0.361084 1.888380 -0.459576
O 0.309021 1.876559 -1.486562
C -0.019165 -0.982670 -1.706587
C 1.196175 -1.547803 -0.965686
O 0.833745 -2.562248 -0.073584
O -0.371450 2.784387 0.356559
C 2.140940 -0.511414 -0.387190
O 1.550647 0.231718 0.662468
C 2.399554 1.274063 1.101239
H -2.169409 -2.276419 -0.613341
H -3.654945 -0.708523 1.141987
H -0.485820 -1.806996 -2.260944
H 0.274447 -0.200704 -2.412653
H 1.789976 -2.038220 -1.759559
H 0.487846 -2.195762 0.764850
H 2.449177 0.180129 -1.195119
H 3.043018 -1.028589 -0.010243
H 1.903490 1.779702 1.937367
H 3.370772 0.872195 1.437140
H 2.566437 2.001684 0.288942
O -0.029275 -1.363096 2.334804
H 0.492693 -0.606263 2.021914
H -0.937759 -1.043303 2.358448

MISO.2H2O
E= -890.649284
N 0.306224 -1.171931 -1.039883
C -0.029248 -2.398108 -0.538684
C -1.375389 -2.356155 -0.250495
N -1.874235 -1.125395 -0.542241
C -0.847685 -0.451138 -1.005330
N -0.964062 0.915631 -1.443282
O -1.935147 1.539119 -1.059546
C 1.688682 -0.771468 -1.299033
C 2.387179 -0.185027 -0.064964
C 2.001356 1.242579 0.261013
O 0.664189 1.296053 0.690425
C 0.273167 2.603407 1.025774
O -0.090955 1.356331 -2.178688
O 2.285677 -1.045411 1.030318
O -0.032680 -0.825504 2.580352
H 0.721358 -3.171062 -0.411685
H -2.002863 -3.146860 0.149011
H 2.213169 -1.692673 -1.583570
H 1.706840 -0.075684 -2.143310
H 3.456141 -0.144043 -0.345681
H 1.455275 -0.884557 1.527214
H 2.146209 1.882620 -0.633160
H 2.668426 1.614669 1.062463
H -0.753777 2.554891 1.405740
H 0.929061 3.024110 1.808385
H 0.299391 3.263566 0.139502
H -0.813945 -0.295999 2.331111
H -0.378608 -1.546601 3.110499
O -2.522605 0.307163 1.845223
H -2.844502 1.210122 1.784701
H -2.613884 -0.047190 0.942273

MISO.3H2O

E= -967.046559
N 0.537332 -1.369661 -0.838253
C -0.005183 -2.478873 -0.256937
C -1.363768 -2.263618 -0.174788
N -1.667675 -1.038510 -0.671183
C -0.514988 -0.532250 -1.049191
N -0.401495 0.771357 -1.636941
O -1.301046 1.565383 -1.403717
C 1.979355 -1.169328 -0.984632
C 2.631993 -0.485553 0.222909
C 2.446585 1.016762 0.283355
O 1.111647 1.344191 0.576603
C 0.933661 2.738497 0.670912
O 0.570051 1.006734 -2.338132
O 2.305634 -1.133670 1.414012
O -0.237786 -0.704202 2.435949
H 0.624244 -3.298167 0.073377
H -2.127582 -2.922683 0.225411
H 2.403107 -2.177428 -1.076385
H 2.173059 -0.618587 -1.910004
H 3.716605 -0.633867 0.064725
H 1.419040 -0.856797 1.735546
H 2.753471 1.468270 -0.682609
H 3.110026 1.418279 1.073503
H -0.084461 2.927026 1.029676
H 1.648836 3.176553 1.389323
H 1.075635 3.220067 -0.313763
H -0.796291 0.050811 2.148577
H -0.313970 -0.728104 3.392074
O -1.939900 1.319132 1.543126
H -1.690438 1.525395 0.631055
H -2.821916 0.922970 1.419064
O -4.117576 -0.032509 0.388312
H -3.401044 -0.331199 -0.204780
H -4.739580 0.440864 -0.169148

MISO.4H2O
E= -1043.440628
C 0.673897 -1.302205 0.422552
N -0.340168 -1.840048 -0.309350
C 0.226286 -2.173636 -1.505615
C 1.562118 -1.843359 -1.421725
N 1.826525 -1.292219 -0.210157
C -1.777955 -1.927894 -0.048439
C -2.552914 -0.651773 -0.400091
O -2.287340 -0.228076 -1.700216
N 0.521463 -0.799948 1.756821
O -0.405530 -1.222373 2.430399
O 1.342656 0.021340 2.138165
C -2.485265 0.454974 0.633994
O -1.205277 1.051273 0.682246
C -1.122197 1.981290 1.747015
O 0.157407 0.897418 -2.334391
O 1.756373 1.937233 -0.175070
O 4.106187 0.457289 -0.162811
H -0.374131 -2.576863 -2.314163
H 2.333393 -1.964000 -2.175493
H -2.140196 -2.733278 -0.699559
H -1.932865 -2.219652 0.994756
H -3.611713 -0.971882 -0.386088
H -1.384221 0.152400 -1.788212
H -2.746355 0.041851 1.628280
H -3.238557 1.223261 0.374734
H -0.149180 2.482158 1.685146
H -1.916128 2.743484 1.661899
H -1.211318 1.467765 2.719312
H 0.797388 1.077489 -1.620987
H -0.159282 1.791625 -2.531478
H 1.538874 1.442835 0.628048
H 2.700096 1.718191 -0.292699
H 3.472967 -0.265094 0.011197
H 4.739612 0.434384 0.558264
H 0.296161 3.295377 -0.813032
O -0.608455 3.399883 -1.144651
H -1.061285 2.649168 -0.736618

MISO.5H2O
E= -1119.839362
N 2.318060 0.174373 -0.758671
C 1.668015 -0.636380 0.046429
N 1.090913 -1.688862 -0.594866
C 1.392181 -1.506505 -1.914593
C 2.157950 -0.365036 -1.994664
N 1.608384 -0.434446 1.466819
O 1.757286 0.708823 1.870318
C 0.172422 -2.723722 -0.116567
C -1.290454 -2.269704 -0.043583
C -1.655290 -1.458042 1.182246
O -1.069300 -0.181812 1.143815
C -1.401158 0.552758 2.303410
O -1.708213 -1.676193 -1.237740
O 1.428303 -1.409096 2.178747
O -0.812848 0.793692 -2.003027
O 0.192087 2.522967 0.097675
O 2.816422 3.013255 -0.479308
O -2.586573 2.694366 0.368875
H 1.004143 -2.176973 -2.674028
H 2.583155 0.094287 -2.880914
H 0.244472 -3.534850 -0.852022
H 0.524251 -3.092117 0.852156
H -1.862928 -3.209884 0.057754
H -1.246942 -0.816603 -1.389988
H -1.332888 -2.002640 2.093529
H -2.758452 -1.361433 1.222727

H -0.940007 1.542918 2.227907
H -2.493182 0.684836 2.382668
H -1.021748 0.042995 3.206938
H -0.459409 1.385507 -1.316040
H -1.761173 1.014677 -2.054049
H 0.602219 1.943281 0.757775
H 0.960214 3.016255 -0.246249
H 2.880489 2.040231 -0.531435
H 3.452998 3.362176 -1.106355
H -1.618729 2.780541 0.278563
H -2.943854 3.561616 0.164094
O -3.579356 0.741710 -1.429505
H -3.353904 -0.161099 -1.169741
H -3.344680 1.306908 -0.671118

[MISO]-
E= -737.904505
N -3.177322 -0.640512 -0.343008
C -2.091882 0.026108 0.045907
N -1.040764 -0.828325 0.339345
C -1.514268 -2.105730 0.091083
C -2.814139 -1.953696 -0.316695
N -1.997363 1.403600 0.132658
O -0.823654 1.923304 0.378956
C 0.287423 -0.571380 0.855221
C 1.335265 -0.351216 -0.236712
O 1.007749 0.680414 -1.115828
O -3.021748 2.116493 -0.044280
C 2.670408 -0.096004 0.435884
O 3.717821 -0.231578 -0.497239
C 4.944830 0.144977 0.045332
H -0.879836 -2.976787 0.232265
H -3.513945 -2.739075 -0.598481
H 0.244304 0.304020 1.514590
H 0.576093 -1.452444 1.452325
H 1.428373 -1.285877 -0.826463
H 0.346894 1.271088 -0.664772
H 2.659868 0.932092 0.852528
H 2.833085 -0.805611 1.277218
H 5.715876 0.007849 -0.724609
H 4.941023 1.206291 0.361144
H 5.209251 -0.472301 0.928299

[MISO]-.H2O
E= -814.310592
C -1.614652 2.293459 0.787806
N -0.250908 2.268238 0.757961
C 0.053724 1.357449 -0.160541
N -1.074286 0.801297 -0.739318
C -2.148760 1.404981 -0.108443
N 1.346776 1.004488 -0.514134
O 2.316184 1.365397 0.232414
C -1.217307 -0.391559 -1.554598
C -1.318563 -1.691618 -0.759064
C -0.164934 -1.921076 0.206065
O -0.443388 -1.196026 1.385159
C 0.699252 -0.977295 2.181692
O 1.527932 0.282612 -1.576082
O -2.558062 -1.798809 -0.077972
H -3.168693 1.136937 -0.368241
H -2.152393 2.968505 1.451409
H -2.146397 -0.279588 -2.135785
H -0.357330 -0.439566 -2.230578
H -1.304164 -2.506144 -1.504823
H -2.431743 -1.341732 0.765875
H 0.785602 -1.593544 -0.251163
H -0.081260 -2.999413 0.448724
H 0.363426 -0.478932 3.099707
H 1.186531 -1.936256 2.440499
H 1.430256 -0.327875 1.670713
H 2.983563 -0.896572 -1.016074
O 3.592297 -1.147142 -0.296688
H 3.529274 -0.318295 0.206013

[MISO]-.2H2O
E= -890.714370
C 1.671347 -2.396010 -0.126703
C 2.362695 -2.014656 -1.245600
N 2.456707 -0.657787 -1.326979
C 1.817649 -0.204377 -0.254446
N 1.317682 -1.229059 0.531655
N 1.682742 1.135271 0.058638
O 1.172906 1.456977 1.216460
C 0.412018 -1.234003 1.674834
C -1.040773 -0.840860 1.374441
O -1.285899 0.531549 1.548138
O 2.074696 2.014729 -0.767627
C -1.474389 -1.296142 -0.004361
O -2.874291 -1.180989 -0.087712
C -3.323430 -1.199057 -1.417290
O -0.173596 3.568269 -0.287615
O -2.431451 1.854553 -0.857512
H 1.392707 -3.368201 0.269782
H 2.809391 -2.658917 -2.000364
H 0.426454 -2.268696 2.048965
H 0.804220 -0.578959 2.461022
H -1.676483 -1.371649 2.108350
H -0.427867 1.003458 1.399848
H -0.988129 -0.659257 -0.769383
H -1.164982 -2.346124 -0.191143
H -4.419716 -1.144917 -1.397848
H -2.930560 -0.329601 -1.972061
H -3.020018 -2.133254 -1.930628
H -2.338602 1.344793 -0.038535

H -1.702686 2.498783 -0.789386
H 0.613739 3.218572 -0.750792
H 0.012992 3.204924 0.590679

[MISO]-.3H2O
E= -967.119585
N -0.551258 1.417751 -0.764985
C -0.237508 2.590693 -0.105009
C 1.103189 2.536276 0.165186
N 1.642056 1.365634 -0.293794
C 0.626061 0.706069 -0.851907
N 0.726996 -0.531070 -1.460516
O 1.730388 -1.284279 -1.162397
C -1.915136 1.054891 -1.108430
C -2.670130 0.232812 -0.054982
C -2.361434 -1.252222 -0.055727
O -1.140170 -1.503231 0.580541
C -0.736688 -2.838760 0.413715
O -0.192034 -0.908559 -2.262345
O -2.599681 0.805278 1.221674
O -0.084466 0.615444 2.522140
H -1.004139 3.324840 0.120845
H 1.716388 3.287893 0.657100
H -2.451492 2.008776 -1.228496
H -1.902267 0.520627 -2.064874
H -3.730418 0.303110 -0.370109
H -1.735410 0.593702 1.640572
H -2.332606 -1.616313 -1.101419
H -3.174241 -1.786057 0.482014
H 0.156876 -2.995863 1.029315
H -1.534442 -3.532223 0.747497
H -0.493451 -3.038654 -0.644856
H 0.475777 -0.140255 2.244920
H 0.304433 1.359605 2.044559
O 1.816119 -1.341459 1.637162
H 1.659671 -1.425288 0.670678
H 2.680059 -0.901797 1.625275
O 4.060678 -0.099734 0.178658
H 3.688740 -0.763770 -0.420917
H 3.375165 0.589233 0.062418

[MISO]-.4H2O
E= -1043.516892
C -2.693170 -2.499328 0.818414
N -3.121898 -1.265133 0.425464
C -2.124271 -0.770590 -0.295579
N -1.064986 -1.654994 -0.396056
C -1.438899 -2.765498 0.335340
N -2.127333 0.484350 -0.878753
O -3.166021 1.217839 -0.780231
C 0.191265 -1.563115 -1.115806
C 1.289345 -0.915830 -0.283079
C 2.538350 -0.761995 -1.114884
O 3.457396 0.073605 -0.442686
C 4.655559 0.218121 -1.147833
O -1.032727 0.925970 -1.434512
O 1.577157 -1.684894 0.857530
O 2.472211 1.887076 1.677626
H -0.761126 -3.604763 0.454676
H -3.311394 -3.138503 1.445301
H 0.495900 -2.594485 -1.359412
H 0.027450 -0.998406 -2.039325
H 0.944131 0.090230 0.006062
H 1.135327 -1.250148 1.615502
H 2.995625 -1.759028 -1.282381
H 2.286915 -0.320686 -2.099753
H 5.309581 0.885201 -0.572534
H 5.162131 -0.756756 -1.278691
H 4.484782 0.659003 -2.148562
H 2.838332 1.302527 0.996708
H 1.634214 2.209203 1.291435
H -0.352846 1.764611 -0.078628
O -0.166568 2.222246 0.777787
H -0.831253 2.932379 0.746285
O 0.489111 0.090052 2.689991
H 1.289516 0.642887 2.618297
H -0.150641 0.631581 2.199709
H -2.686828 2.879496 -0.493418
O -2.266731 3.765847 -0.358357
H -1.730002 3.843575 -1.152464

[MISO]-.5H2O
E= -1119.915044
N 1.201717 2.156079 0.153546
C 1.423185 0.933695 0.640977
N 2.221921 0.187733 -0.201741
C 2.513503 1.011353 -1.275542
C 1.885805 2.197881 -1.032067
N 0.964940 0.467999 1.858228
O 0.030748 1.116410 2.460692
C 2.564593 -1.221560 -0.195905
C 1.492606 -2.153138 -0.780599
C 0.403241 -2.572837 0.191111
O -0.578734 -1.571130 0.316505
C -1.367591 -1.758692 1.476291
O 0.983054 -1.687624 -2.002279
O 1.475325 -0.597226 2.350001
O -0.908070 0.583004 -1.818988
O -2.033555 1.370429 0.794032
O -1.108690 3.453887 -0.887780
O -2.734441 -1.606234 -1.635096
H 3.099886 0.648383 -2.113033
H 1.872361 3.087683 -1.657514
H 3.461201 -1.309104 -0.828406

H 2.808943 -1.522774 0.828608
H 2.036000 -3.088529 -1.015898
H 0.457152 -0.874980 -1.858617
H 0.859553 -2.776039 1.176987
H -0.075287 -3.503902 -0.177969
H -2.156623 -0.999502 1.465149
H -1.829854 -2.764436 1.467501
H -0.746862 -1.628653 2.377540
H -1.032616 0.661074 -0.855126
H -1.624091 -0.033037 -2.058538
H -1.304362 1.304434 1.470297
H -1.908212 2.234362 0.364496
H -0.316018 3.252667 -0.348790
H -1.083077 2.740939 -1.545138
H -3.358305 -1.160655 -1.031697
H -1.966621 -1.792034 -1.070026
O -4.254766 -0.044811 0.206835
H -3.489721 0.511005 0.489714
H -4.882519 0.576565 -0.166748

[MISO-NO2], I
E= -532.768435
C -2.072890 0.931379 -0.294726
C -3.435344 0.811471 -0.349035
N -3.830893 -0.474138 -0.054664
C -2.723944 -1.125934 0.177474
N -1.603083 -0.327687 0.046415
C -0.301907 -0.740664 0.204960
C 0.829220 0.165693 -0.087218
O 0.839498 1.234910 0.853278
C 2.135226 -0.594528 -0.041546
O 3.165780 0.322651 -0.304504
C 4.439337 -0.262356 -0.206489
H -1.402070 1.774500 -0.411597
H -4.160350 1.587890 -0.577522
H -0.161765 -1.759859 0.566423
H 0.714743 0.587080 -1.109396
H 1.665132 1.717769 0.716224
H 2.265623 -1.043931 0.964295
H 2.130758 -1.410366 -0.791774
H 5.182535 0.514578 -0.418365
H 4.616196 -0.665555 0.806897
H 4.557820 -1.082613 -0.937311
H -2.631201 -2.177573 0.439166

[MISO-NO2], II
E= -532.743313
C -2.321661 0.943783 0.335597
C -3.510345 0.613477 -0.262784
N -3.531377 -0.718586 -0.599383
C -2.371252 -1.185094 -0.213639
N -1.593013 -0.222979 0.370267
C -0.255784 -0.392421 0.856247
C 0.830573 0.106221 -0.141880
O 0.675007 1.453382 -0.199144
C 2.197933 -0.305553 0.371365
O 3.145329 0.072273 -0.585858
C 4.454007 -0.232308 -0.180006
H -1.920174 1.875199 0.722529
H -4.355217 1.264688 -0.469634
H -0.076959 -1.464159 1.026660
H -0.128449 0.143205 1.807883
H 0.620200 -0.375525 -1.120121
H 2.389998 0.200928 1.340085
H 2.230396 -1.403299 0.539637
H 5.133567 0.085507 -0.978761
H 4.717740 0.300361 0.751764
H 4.578842 -1.318219 -0.011357
H -2.022729 -2.211604 -0.314257

[MISO-NO2], III
E= -532.732894
C -3.536024 0.639276 -0.253201
N -3.625816 -0.700633 -0.608526
C -2.494240 -1.191097 -0.259258
N -1.637534 -0.312144 0.315352
C -2.318460 0.900130 0.316406
C -0.295719 -0.580408 0.784566
C 0.774987 0.023806 -0.098333
C 2.139275 -0.515164 0.267439
O 3.082393 0.160481 -0.525104
C 4.402826 -0.206868 -0.216331
O 0.732242 1.423949 0.079195
H -1.857695 1.804677 0.700029
H -4.357816 1.326779 -0.433718
H -0.178430 -1.672892 0.824005
H -0.181011 -0.182169 1.804997
H 0.561079 -0.236011 -1.153669
H 1.496388 1.795246 -0.378177
H 2.339831 -0.332105 1.343915
H 2.185699 -1.607695 0.086052
H 5.071691 0.365020 -0.869448
H 4.644194 0.020825 0.837567
H 4.566579 -1.285730 -0.389776

[MISO-OH], I
E= -662.082834
C -1.208026 -0.060030 -0.576281
N -0.109066 -1.038151 -0.368153
C 1.172550 -0.572204 -0.089213
N 1.407114 0.774506 -0.001214
C 0.412939 1.802654 -0.221429
C -0.926705 1.232321 0.185893
C 2.750559 0.904452 0.271228

C 3.238245 -0.379162 0.332177
N 2.249814 -1.297149 0.107460
O -0.326448 -2.259634 -0.601149
O -1.912033 2.184151 -0.112361
O -2.442691 -0.599971 -0.257481
C -2.600090 -1.080864 1.070674
H 3.214621 1.877792 0.395105
H 4.259559 -0.690168 0.534174
H 0.378578 2.105520 -1.281128
H 0.655981 2.687667 0.380584
H -1.263490 0.169342 -1.656279
H -1.803154 -1.787597 1.339433
H -2.638352 -0.257862 1.803334
H -3.559421 -1.608077 1.087366
H -2.769384 1.740022 -0.110099
H -0.897434 1.009559 1.270499

[MISO-OH], II
E= -662.077892
C -1.392724 0.577578 0.031843
N -1.603132 -0.750113 -0.179999
C -2.901536 -0.865640 -0.618775
C -3.369310 0.432335 -0.661983
N -2.419677 1.339312 -0.252794
C -0.507453 -1.580970 0.272602
C 0.615219 -0.556905 0.522372
O 1.203979 -0.784889 1.749500
N -0.116988 0.760991 0.525621
O 0.391477 1.819678 0.970706
C 1.636872 -0.578080 -0.604555
O 2.681549 0.285960 -0.281311
C 3.673767 0.322197 -1.276325
H -3.361252 -1.815306 -0.868662
H -4.355554 0.761047 -0.979240
H -0.745635 -2.093909 1.215648
H 2.007404 -1.619519 -0.710044
H 1.143622 -0.289345 -1.555488
H 4.456532 1.010303 -0.939314
H 4.114010 -0.678999 -1.436080
H 3.261460 0.686276 -2.233830
H -0.221832 -2.326463 -0.482374
H 1.934782 -0.154508 1.837355

[MISO-OH], III
E= -662.076957
O -0.158980 1.827091 -0.095557
N 1.090198 1.659727 -0.652324
C 1.690958 0.520959 -0.296529
N 1.156642 -0.419125 0.562217
C -0.115490 -0.226429 1.211642
C -0.929593 0.667123 0.299914
C 2.079119 -1.418339 0.679199
C 3.141380 -1.037579 -0.134783
N 2.908073 0.144720 -0.733320
C -1.393122 -0.021498 -0.986327
O -1.947270 -1.289962 -0.781525
C -3.269497 -1.294470 -0.289219
H 1.912347 -2.290975 1.303429
H 4.061609 -1.591469 -0.304583
H -0.002430 0.275254 2.184059
H -0.623307 -1.189892 1.350844
H -2.108405 0.659164 -1.490132
H -0.535688 -0.152567 -1.660535
H -3.321222 -1.016262 0.775607
H -3.905107 -0.598869 -0.866289
H -3.656147 -2.312666 -0.415709
O -1.975084 1.162551 1.060102
H -2.477901 1.784987 0.519760

[MISO-OH], IV
E= -662.020532
N -1.249704 -0.840307 0.049796
C -2.185614 -1.851305 0.054476
C -3.414261 -1.249345 -0.125606
N -3.292185 0.090744 -0.237930
C -1.997124 0.339619 -0.128842
N -1.342724 1.565077 -0.080398
O -2.042428 2.541097 -0.295718
C 0.095809 -1.050757 0.142062
C 1.132601 0.019520 0.072858
C 2.496700 -0.626299 -0.010375
O 3.434848 0.337769 -0.379897
C 4.743678 -0.166325 -0.360077
O 1.102898 0.846168 1.215178
H -1.888517 -2.888835 0.169188
H -4.383125 -1.740917 -0.172203
H 0.370082 -2.091975 0.328414
H 0.981262 0.623105 -0.842968
H 0.399411 1.495548 1.059208
H 2.745100 -1.064348 0.979221
H 2.472578 -1.453237 -0.752205
H 5.417002 0.641658 -0.667583
H 5.026241 -0.504037 0.653736
H 4.858981 -1.016482 -1.058570

[MISO-OH], V
E= -662.001513
C -1.002010 2.058412 -0.289842
C -2.081132 1.601011 -1.014304
N -2.294522 0.283038 -0.771357
C -1.370473 -0.050662 0.093553
N -0.543814 0.993947 0.446971
N -1.153823 -1.403561 0.536390
O -0.255092 -1.595408 1.351546

C 0.573487 1.029070 1.292628
C 1.886852 1.448673 0.743853
H 1.774897 2.236839 -0.016581
O -1.861643 -2.266719 0.061911
C 2.572257 0.244308 0.103536
O 1.760429 -0.199706 -0.951859
C 2.135962 -1.471556 -1.419786
H -0.544590 3.038526 -0.200169
H -2.712763 2.164714 -1.695081
H 0.504896 0.424719 2.192568
H 2.529109 1.839351 1.546630
H 2.692078 -0.559539 0.857036
H 3.581792 0.516574 -0.264380
H 1.468359 -1.731232 -2.249564
H 3.179914 -1.475481 -1.782541
H 2.032216 -2.229314 -0.622554

[MISO-CH2NO2]-
E= -493.620721
C 2.254678 1.100471 -0.005540
N 1.114143 0.348709 0.131468
C 1.492545 -0.954807 0.039296
N 2.795309 -1.082684 -0.155405
C 3.277712 0.200173 -0.183462
C -0.233625 0.857196 0.376318
C -1.321731 -0.028094 -0.235696
C -2.670128 0.735115 -0.130043
O -3.690177 -0.221463 -0.216688
O -1.439629 -1.230857 0.356271
H -2.787661 1.489575 -0.931762
H -2.714386 1.267482 0.849840
H -0.271545 1.881101 -0.039140
H -0.411053 0.906242 1.464990
H -1.071719 -0.090940 -1.339006
H -3.164172 -1.021690 0.063960
H 2.227004 2.186113 0.028213
H 4.334766 0.411728 -0.336505
H 0.734346 -1.733538 0.125066

[MISO-CH2NO2]-.H2O
E= -570.027802
C -3.698656 0.108227 -0.287234
C -2.821784 -0.921252 -0.045152
N -1.598972 -0.331662 0.164783
C -1.790289 1.010936 0.049979
N -3.048071 1.313054 -0.227396
C -0.361369 -1.033826 0.496998
C 0.882760 -0.390951 -0.130782
O 1.110256 0.841068 0.388991
C 2.046094 -1.368699 0.079396
O 3.222606 -0.997634 -0.597355
H 1.778886 -2.375877 -0.294433
H 2.224402 -1.445637 1.175266
H -0.496222 -2.074824 0.157342
H -0.227964 -1.031113 1.593052
H 0.694324 -0.381018 -1.243434
H 3.430647 -0.071131 -0.360005
H -2.945508 -1.999982 -0.009754
H -4.762686 0.041556 -0.507505
H -0.942222 1.681719 0.175264
H 2.478077 1.437329 0.255993
O 3.459464 1.739087 0.109627
H 3.418408 2.262464 -0.692832

[MISO-CH2NO2]-.2H2O
E= -646.433510
C 3.863912 0.281867 0.353447
C 3.093507 -0.852202 0.425133
N 1.824255 -0.467136 0.066054
C 1.886644 0.864366 -0.209198
N 3.103288 1.351110 -0.041798
C 0.679649 -1.365496 -0.044636
C -0.669298 -0.657666 -0.046330
O -0.911843 0.035952 -1.195294
C -1.765115 -1.739270 0.138867
O -2.954512 -1.241610 -0.422366
H -1.929320 -1.975769 1.204629
H -1.460916 -2.675671 -0.379720
H 0.741072 -2.075844 0.799792
H 0.758013 -1.934729 -0.987052
H -0.697953 -0.000089 0.862423
H -2.565028 -0.595956 -1.070385
H 3.317576 -1.879569 0.698605
H 4.925153 0.384256 0.572429
H 1.004738 1.412571 -0.531069
O -3.543250 1.051651 1.310179
H -2.882322 1.609674 0.861566
H -3.498579 0.225355 0.802163
O -1.532837 2.373342 -0.310728
H -1.287141 1.482602 -0.740207
H -1.914345 2.891073 -1.020995

[MISO-CH2NO2]-.3H2O
E= -722.836682
C 2.866578 -0.895371 1.050147
C 1.712143 -1.627820 1.178580
N 1.036475 -1.474731 -0.008900
C 1.789218 -0.649519 -0.779024
N 2.909534 -0.288657 -0.179576
C -0.326266 -1.899515 -0.287999
C -1.296956 -0.729790 -0.126948
O -1.072328 0.244013 -1.046681
C -2.748300 -1.223130 -0.256023
O -3.547946 -0.097262 -0.530197

H -3.108088 -1.709376 0.667205
H -2.818385 -1.959618 -1.087276
H -0.562289 -2.736591 0.390969
H -0.378974 -2.256658 -1.328634
H -1.168010 -0.364382 0.930153
H -2.891389 0.455292 -1.022630
H 1.311508 -2.235696 1.984311
H 3.668900 -0.776309 1.774358
H 1.442237 -0.314859 -1.752624
O -2.642421 2.039129 1.489876
H -1.761600 2.180115 1.102299
H -3.036172 1.355536 0.927636
O 0.101516 2.096034 0.293521
H -0.328323 1.395715 -0.312709
H 0.558582 1.580528 0.964849
O 2.738052 2.655902 -0.871112
H 3.047842 1.755605 -0.690743
H 1.808606 2.622745 -0.583498

[MISO-CH2NO2]-.4H2O
E= -799.242474
C -3.339005 -0.094824 -0.330902
C -2.277299 -0.149805 0.543519
N -1.535394 -1.234031 0.146115
C -2.171977 -1.766996 -0.931711
N -3.267226 -1.112245 -1.250850
C -0.287756 -1.703402 0.723451
C 0.939975 -0.908106 0.290185
O 1.021224 0.327742 0.872351
C 2.189707 -1.728160 0.669333
O 3.295893 -0.857973 0.723459
H 2.392144 -2.522753 -0.068563
H 2.033103 -2.206538 1.660514
H -0.178658 -2.764083 0.437599
H -0.366458 -1.645525 1.820695
H 0.907754 -0.837876 -0.826837
H 2.848928 -0.020812 0.987525
H -1.967422 0.480117 1.376762
H -4.155254 0.624145 -0.340364
H -1.778834 -2.644226 -1.442403
O 3.390829 0.157970 -2.036431
H 2.691316 0.810888 -1.867790
H 3.518733 -0.249687 -1.164898
O 1.371833 2.032576 -0.983708
H 1.238314 1.370653 -0.208546
H 1.904406 2.738165 -0.609497
O -0.601318 2.159180 2.167175
H -0.892546 2.574140 1.340178
H -0.001491 1.453685 1.843655
O -1.319345 2.788156 -0.663342
H -1.775343 1.939290 -0.722002
H -0.395154 2.570885 -0.902159

[MISO-CH2NO2]-.5H2O
E= -875.637868
C -3.161790 -1.352475 0.202036
C -2.027979 -2.058704 0.530276
N -1.043505 -1.603980 -0.306849
C -1.608813 -0.646982 -1.082199
N -2.889397 -0.470294 -0.812036
C 0.364003 -1.987036 -0.284765
C 1.262333 -0.866654 0.243551
O 0.992722 -0.533308 1.535783
C 2.718335 -1.311690 0.074747
O 3.164476 -1.313798 -1.268137
H 2.840985 -2.336559 0.469575
H 3.335101 -0.646307 0.705134
H 0.672507 -2.283199 -1.299587
H 0.449750 -2.854894 0.387479
H 1.122131 -0.010020 -0.472282
H 3.284835 -0.373954 -1.491999
H -1.822905 -2.816277 1.280091
H -4.154353 -1.434380 0.638173
H -1.039902 -0.092737 -1.825266
O 3.492131 1.528179 -1.110377
H 2.906463 1.712692 -0.332893
H 4.372867 1.730039 -0.788375
O 1.932795 1.957615 1.100616
H 1.645193 1.058726 1.427735
H 1.101155 2.360335 0.802467
O -0.808496 2.762715 0.334878
H -1.023558 2.005457 0.946376
H -1.072095 3.544865 0.825021
O -1.187396 0.733487 2.017263
H -0.341979 0.177345 1.854230
H -1.909645 0.150214 1.760376
O -2.929898 2.361845 -1.686679
H -3.179454 1.453508 -1.451284
H -2.183992 2.542437 -1.091416

[MISO-C2H4O2]-, I
E= -509.001491
C -0.481803 2.336766 0.135282
N 0.740862 1.745460 0.233468
C 0.526200 0.456309 -0.046740
N -0.808085 0.211572 -0.346699
C -1.453726 1.430568 -0.204547
N 1.497331 -0.527589 -0.059783
C -1.529488 -1.038118 -0.512449
C -1.810466 -1.716507 0.815554
H -2.417068 -2.623678 0.666825
H -0.857978 -2.006742 1.279816
H -2.352441 -1.039536 1.494575
H -2.523178 1.523639 -0.376912

H -0.607886 3.405428 0.307790
H -0.934167 -1.697004 -1.151815
H -2.471877 -0.781804 -1.026028
O 2.680480 -0.234197 0.284986
O 1.150712 -1.715868 -0.423206

[MISO-C2H4O2]-, II
E= -509.076599
N 2.570061 0.576759 -0.125457
C 1.234105 0.795293 0.125319
N 0.567950 -0.455146 0.166452
C 1.495432 -1.408244 -0.079133
C 2.711015 -0.743898 -0.257694
C -0.809050 -0.703370 0.570733
C -1.865733 -0.127111 -0.401356
O -1.974652 1.174186 -0.372533
N 0.680290 1.961373 0.280688
H -1.646448 -0.577242 -1.416357
O -3.108117 -0.798867 0.034787
H 1.242120 -2.465691 -0.093472
H 3.673879 -1.204245 -0.480759
H -0.989966 -0.238014 1.554099
H -0.924409 -1.794151 0.661336
H -3.659850 -0.023655 0.193602
H -0.355890 1.843533 0.284511

[MISO-C2H4O2]-.H2O
E= -585.409780
C 2.548012 -0.389919 -0.222554
C 2.262142 -1.689431 0.109009
N 0.919406 -1.888948 0.223456
C 0.371273 -0.702111 -0.037451
N 1.327655 0.254777 -0.338592
N -0.985446 -0.439974 -0.028600
O -1.378727 0.747854 -0.365939
C 1.217466 1.694523 -0.513169
C 1.074938 2.421030 0.810468
O -1.794969 -1.361611 0.306987
H 1.061020 3.510504 0.653549
H 0.130064 2.126558 1.286866
H 1.910826 2.174866 1.483727
H 3.480835 0.138425 -0.401769
H 2.969481 -2.502923 0.264609
H 0.355842 1.901253 -1.154567
H 2.137561 2.008214 -1.033600
H -3.708832 -0.591038 0.175527
O -4.158645 0.239039 -0.052433
H -3.352367 0.746351 -0.254934

[MISO-C2H4O2]-.2H2O
E= -661.815947
C -2.450505 1.013714 -0.235657
C -1.663721 2.112464 -0.016032
N -0.346786 1.766179 0.047630
C -0.322055 0.444133 -0.131437
N -1.591238 -0.067497 -0.329911
N 0.819507 -0.333288 -0.135107
O 0.694910 -1.595131 -0.379120
C -2.068348 -1.441280 -0.402337
C -2.160381 -2.085371 0.967025
O 1.951250 0.204003 0.104917
H -2.583866 -3.097895 0.888292
H -1.155762 -2.162742 1.403746
H -2.799656 -1.490171 1.637225
H -3.523750 0.888197 -0.348176
H -1.981824 3.147295 0.092563
H -1.392935 -2.008729 -1.048463
H -3.061016 -1.390209 -0.878343
H 3.468149 -1.383548 0.108873
O 3.416601 -2.337416 -0.044691
H 2.458301 -2.382860 -0.218265
H 2.542987 2.173308 0.234702
O 2.256084 3.097633 0.284596
H 1.298284 2.934932 0.234578

[MISO-C2H4O2]-.3H2O
E= -738.216777
C 1.228229 -2.386041 -0.225029
C -0.107202 -2.547764 0.024493
N -0.740937 -1.339228 0.083581
C 0.215566 -0.431488 -0.127959
N 1.442767 -1.023340 -0.342057
N 0.008098 0.932652 -0.145534
O 1.010240 1.703808 -0.384972
C 2.773148 -0.443129 -0.468666
C 3.358250 -0.063744 0.877116
O -1.164523 1.387178 0.091941
H 4.385125 0.312006 0.756965
H 2.749827 0.729444 1.331536
H 3.380861 -0.932196 1.553100
H 2.044181 -3.093349 -0.342857
H -0.654906 -3.477112 0.163209
H 2.707042 0.432991 -1.119774
H 3.389844 -1.210348 -0.963404
H -0.970839 3.596451 0.446265
O -0.202326 4.175461 0.355715
H 0.434314 3.505786 0.046331
H -2.556992 0.634026 -0.886460
O -3.141099 -0.026556 -1.313118
H -2.606911 -0.821397 -1.183604
H -2.516222 -1.254137 1.172456
O -3.472166 -1.292540 1.346003
H -3.803780 -0.758581 0.610018

[MISO-C2H4O2]-.4H2O
E= -814.621000
C -2.398667 0.067184 -1.220322
C -1.726472 1.230600 -1.486263
N -0.404120 1.108428 -1.164566
C -0.271664 -0.134981 -0.701545
N -1.462702 -0.825016 -0.734365
N 0.910871 -0.688531 -0.249874
O 0.964372 -1.953869 -0.054804
C -1.828333 -2.083885 -0.090978
C -1.915411 -1.941209 1.415080
O 1.919169 0.084718 -0.031958
H -2.274436 -2.879125 1.863667
H -0.923602 -1.718255 1.829928
H -2.595768 -1.124512 1.695560
H -3.440289 -0.218270 -1.332687
H -2.123727 2.150872 -1.907540
H -1.089975 -2.840886 -0.371532
H -2.804832 -2.361169 -0.518115
H 3.647290 -1.185352 0.005934
O 3.827213 -2.136618 -0.008368
H 2.905316 -2.443592 0.005028
H 2.356686 2.198827 -0.676947
O 1.761239 2.947360 -0.816121
H 0.950484 2.451861 -1.055966
H -1.978008 1.596143 0.939078
O -1.823843 1.652944 1.888652
H -0.854606 1.773627 1.950835
O 1.006302 2.000477 1.843428
H 1.157122 2.649570 1.136160
H 1.367654 1.199752 1.415767

[MISO-C2H4O2]-.5H2O
E= -891.023462
C 1.776665 -2.414587 -0.393012
C 0.415729 -2.542427 -0.375569
N -0.183173 -1.314980 -0.307656
C 0.820748 -0.435687 -0.275537
N 2.045898 -1.058586 -0.345264
N 0.662096 0.932809 -0.194444
O 1.700305 1.681132 -0.231415
C 3.395940 -0.526296 -0.196448
C 3.751732 -0.281807 1.255947
O -0.524399 1.423893 -0.087745
H 4.793508 0.059411 1.341896
H 3.098501 0.496114 1.672604
H 3.635780 -1.202298 1.847786
H 2.580258 -3.143247 -0.441553
H -0.178515 -3.452776 -0.405575
H 3.468349 0.398992 -0.774381
H 4.063832 -1.278234 -0.644624
H -0.281584 3.967599 -0.019475
O 0.609945 4.320125 -0.105143
H 1.116071 3.487883 -0.162448
H -1.712386 0.838887 -1.393958
O -2.245881 0.201899 -1.911823
H -1.801809 -0.619960 -1.658573
H -1.951342 -1.764794 0.410957
O -2.750170 -2.106100 0.849338
H -3.469846 -1.564175 0.476257
O -4.417406 0.052135 -0.081261
H -3.818410 0.161718 -0.844270
H -3.922758 0.490500 0.634519
O -2.398465 0.652983 1.896882
H -1.678889 0.921826 1.291535
H -2.370819 -0.315847 1.877904

[MISO-C2H4O2]
E= -508.958388
C -0.081223 2.386896 0.148447
N 1.014662 1.593946 0.232559
C 0.571206 0.387055 -0.024304
N -0.770937 0.339433 -0.287323
C -1.192610 1.632174 -0.169494
N 1.428805 -0.774701 -0.040969
C -1.678636 -0.782536 -0.558373
C -2.127799 -1.461994 0.716770
H -2.847022 -2.257710 0.482238
H -1.274505 -1.914243 1.237956
H -2.615870 -0.742891 1.390184
H -2.234489 1.897693 -0.325647
H -0.025393 3.458914 0.314975
H -1.171005 -1.481477 -1.229269
H -2.531263 -0.345913 -1.096611
O 2.612232 -0.601231 0.147516
O 0.893544 -1.860605 -0.245262

CH2NO2
E= -244.213341
O 0.667560 1.091180 -0.000098
N 0.105020 0.000000 -0.000009
O 0.667560 -1.091180 -0.000098
C -1.304952 0.000000 0.000189
H -1.793196 0.971168 0.000248
H -1.793196 -0.971168 0.000250

CH3OH
E= -115.616297
C -0.659513 -0.020773 0.000000
O 0.743804 0.122224 0.000000
H -1.084684 0.990482 -0.000001
H -1.028233 -0.547096 0.896804
H -1.028233 -0.547097 -0.896804
H 1.147802 -0.749444 0.000000

CH4
E= -40.430281
C -0.000000 -0.000000 0.000000
H 0.895018 -0.000000 -0.632875
H -0.895018 0.000000 -0.632875
H -0.000000 0.895019 0.632874
H 0.000000 -0.895019 0.632874

CO2
E= -188.516587 in
O 0.000000 0.000000 1.162283
C 0.000000 0.000000 0.000000
O 0.000000 0.000000 -1.162283

CO
E= -113.262326
O 0.000000 0.000000 0.485799
C 0.000000 0.000000 -0.647731

H2O
E= -76.385190
O 0.000000 0.117431 0.000000
H 0.760998 -0.469725 0.000000
H -0.760998 -0.469725 0.000000

NO2-
E= -205.085800
N 0.000000 0.458967 0.000000
O 1.064536 -0.200854 0.000000
O -1.064536 -0.200742 0.000000

NO2-.H2O
E= -281.497056
N -1.282235 0.000954 -0.000968
O -0.622499 1.066584 0.000436
O -0.625308 -1.066412 0.000563
H 1.372256 0.720579 0.000235
O 2.027068 -0.001072 -0.000208
H 1.369293 -0.720063 0.000211

NO2-.2H2O
E= -357.898707
O -0.701767 -1.055690 -0.139429
N -1.118181 0.130316 0.011235
O -2.339729 0.275309 0.091130
O 1.358425 1.762842 -0.031627
O 1.991288 -1.104317 -0.016122
H 1.828971 0.919818 -0.121657
H 0.433243 1.451041 0.005520
H 0.999101 -1.105972 -0.126283
H 2.100213 -1.202251 0.932162

NO2-.3H2O
E= -434.303955
N 0.233413 -0.739600 0.012989
O 0.374410 0.510971 -0.117893
O 1.270499 -1.405553 0.100794
H 2.955769 -0.238346 0.057417
O 3.278276 0.675923 -0.014039
H 2.410104 1.098981 -0.102339
H -2.133644 1.871640 0.930344
O -2.077878 1.703848 -0.012904
H -1.178768 1.298867 -0.116639
H -2.796143 -0.192905 -0.122172
O -2.735450 -1.157579 -0.046889
H -1.770059 -1.281930 -0.010090

NO2-.4H2O
E= -510.706030
O -0.654725 1.309148 1.066328
O 1.128368 0.866480 -0.963588
N 2.265532 0.396493 -0.673517
O 2.332327 -0.244725 0.388129
O -0.932398 -1.197931 -1.312047
O -3.096350 0.420409 -0.269194
H 0.009634 1.135935 0.348453
H -0.213912 1.916595 1.663624
H -0.260756 -0.509488 -1.449803
H -0.687558 -1.534504 -0.429549
H -2.503177 -0.185922 -0.748424
H -2.465459 0.917776 0.270683
O -0.033123 -1.492094 1.390861
H -0.524187 -0.670976 1.550203
H 0.833902 -1.135172 1.105511

NO2-.5H2O
E= -587.109220
O 2.409463 -0.126851 1.048009
O -0.044093 -1.520754 1.640761
O -0.933225 1.210362 1.140042
N 0.048746 1.488310 0.406380
O -0.214605 1.727606 -0.783642
O -2.741745 -0.237815 -0.445989
O -0.396244 -1.754002 -1.152536
H 1.842736 0.664265 0.998487
H 1.765973 -0.795084 1.349136
H -0.389394 -0.615294 1.718694
H -0.164590 -1.719823 0.688855
H -1.178226 -1.174276 -1.166687
H 0.338664 -1.205339 -1.489663
H -2.838067 -0.809415 0.321750
H -2.207608 0.504626 -0.087998
O 1.802610 -0.029151 -1.777991

H 1.230253 0.745531 -1.627239
H 2.201747 -0.168531 -0.899223

OH-
E= -75.764443
O 0.000000 0.000000 0.107006
H 0.000000 0.000000 -0.856050

OH-.H2O
E= -152.193582
O -1.212499 0.095296 0.059882
H -1.492488 -0.619313 -0.515071
H -0.000161 -0.000069 0.072013
O 1.212514 -0.095287 0.059884
H 1.492536 0.619311 -0.515068

OH-.2H2O
E= -228.608038
O -0.050906 1.366295 0.109414
H -0.056657 1.937823 -0.660584
H -0.985742 0.150416 -0.087869
O -1.526528 -0.730770 -0.109464
H -1.689919 -0.889231 0.821825
H 1.153124 0.214201 0.050551
O 1.659579 -0.655679 0.004769
H 0.922036 -1.251968 -0.161678

OH-.3H2O
E= -305.020591
O 0.003622 -0.001354 1.449082
H 0.009767 -0.002338 2.405230
H 1.299734 -0.206315 0.359539
O 1.745147 -0.376759 -0.517553
H 1.079347 -0.948310 -0.920987
H -0.827750 -1.023489 0.363377
O -1.201826 -1.322126 -0.512487
H -1.364357 -0.458718 -0.913721
O -0.547667 1.700286 -0.511684
H -0.470818 1.228192 0.364452
H 0.279868 1.410605 -0.916756

OH-.4H2O
E= -381.429086
O 0.000785 -1.504856 -0.494266
O -0.000335 -0.208030 1.902448
O 2.129731 -0.018433 -0.584395
O -2.129008 -0.019543 -0.585007
H 0.001130 -2.440402 -0.695453
H 1.366499 -0.678254 -0.667670
H 2.262965 -0.005484 0.368380
H -1.365525 -0.679108 -0.667274
H -2.265997 -0.009093 0.367241
H 0.000757 -0.839754 1.139540
H -0.000725 0.648157 1.448363
O -0.000867 1.910087 -0.268052
H 0.765457 1.365492 -0.530014
H -0.767013 1.364645 -0.528941

OH-.5H2O
E= -457.835201
O 0.295609 -0.724653 -1.274361
O -0.129738 -1.279046 1.394550
O -1.315844 1.395673 1.077160
O 0.916645 1.802846 -0.635469
O 2.545253 -0.485784 0.318928
H 0.439505 -1.218654 -2.082649
H 0.025752 -1.283087 0.422398
H -0.491305 -0.386240 1.533653
H -1.830727 0.894421 0.418973
H -0.541476 1.693628 0.558974
H 0.625277 0.963010 -1.079427
H 1.672182 1.466894 -0.131829
H 2.019803 -0.738277 1.090958
H 1.882073 -0.645909 -0.395997
H -1.372571 -0.610758 -1.088318
O -2.325941 -0.574701 -0.779652
H -2.316376 -1.209705 -0.055990
